# Head-centric computing for vestibular stimulation under head-free conditions

**DOI:** 10.3389/fbioe.2023.1296901

**Published:** 2023-12-07

**Authors:** Barbara La Scaleia, Claudia Brunetti, Francesco Lacquaniti, Myrka Zago

**Affiliations:** ^1^ Laboratory of Visuomotor Control and Gravitational Physiology, IRCCS Santa Lucia Foundation, Rome, Italy; ^2^ Laboratory of Neuromotor Physiology, Istituto di Ricovero e Cura a Carattere Scientifico—Scientific Institute for Research, Hospitalization and Healthcare, Santa Lucia Foundation, Rome, Italy; ^3^ Department of Systems Medicine and Centre of Space Bio-medicine, University of Rome Tor Vergata, Rome, Italy

**Keywords:** personalized vestibular tests, unrestrained movements, vestibulopathies, ageing, Stewart platform, semicircular canals, otoliths

## Abstract

**Background:** The vestibular end organs (semicircular canals, saccule and utricle) monitor head orientation and motion. Vestibular stimulation by means of controlled translations, rotations or tilts of the head represents a routine manoeuvre to test the vestibular apparatus in a laboratory or clinical setting. In diagnostics, it is used to assess oculomotor postural or perceptual responses, whose abnormalities can reveal subclinical vestibular dysfunctions due to pathology, aging or drugs.

**Objective:** The assessment of the vestibular function requires the alignment of the motion stimuli as close as possible with reference axes of the head, for instance the cardinal axes naso-occipital, interaural, cranio-caudal. This is often achieved by using a head restraint, such as a helmet or strap holding the head tightly in a predefined posture that guarantees the alignment described above. However, such restraints may be quite uncomfortable, especially for elderly or claustrophobic patients. Moreover, it might be desirable to test the vestibular function under the more natural conditions in which the head is free to move, as when subjects are tracking a visual target or they are standing erect on the moving platform. Here, we document algorithms that allow delivering motion stimuli aligned with head-fixed axes under head-free conditions.

**Methods:** We implemented and validated these algorithms using a MOOG-6DOF motion platform in two different conditions. 1) The participant kept the head in a resting, fully unrestrained posture, while inter-aural, naso-occipital or cranio-caudal translations were applied. 2) The participant moved the head continuously while a naso-occipital translation was applied. Head and platform motion were monitored in real-time using Vicon.

**Results:** The results for both conditions showed excellent agreement between the theoretical spatio-temporal profile of the motion stimuli and the corresponding profile of actual motion as measured in real-time.

**Conclusion:** We propose our approach as a safe, non-intrusive method to test the vestibular system under the natural head-free conditions required by the experiential perspective of the patients.

## 1 Introduction

Vestibular information plays a crucial role for our sense of spatial orientation and postural control. It is fundamental to encode self-motion information (heading direction and speed) (Angelaki and Cullen, 2008; Cullen, 2011; 2012), and to disambiguate visual object motion from self-motion (MacNeilage et al., 2012). In each ear, the vestibular end organs include the 3 semicircular canals, sensing angular accelerations, and the 2 otolith organs (saccule and utricle), sensing linear accelerations. Over the last several years, different methods have been used for stimulating the vestibular system in both basic science and clinical applications (Ertl and Boegle, 2019; Diaz-Artiles and Karmali, 2021). These methods range from galvanic or caloric vestibular stimulation to passive full-body accelerations. These last methods allow more naturalistic vestibular stimulation to the extent that they simulate the conditions of everyday life (Lacquaniti et al., 2023). Since other sources of sensory information in addition to the vestibular ones potentially contribute to passive self-motion perception, appropriate measures are generally taken to minimize non-vestibular cues, for instance by masking visual and auditory cues and by minimizing somatosensory cues.

In diagnostics, controlled translations, rotations or tilts of the head are used to assess oculomotor postural or perceptual responses (e.g., Merfeld et al., 2010). The vestibulo-ocular reflex (VOR) tends to stabilize the gaze during head motion, by producing eye movements in the direction opposite to that of head motion. On the other hand, perceptual tests can assess vestibular function independently of ocular motor responses. Thus, these tests can determine the subjective thresholds for detection or discrimination of head and body motion. Abnormal thresholds can reveal subclinical vestibulopathies or vestibular hypofunction due to aging or drugs (Valko et al., 2012; Kobel et al., 2021b; Diaz-Artiles Karmali, 2021).

**FIGURE 1:**
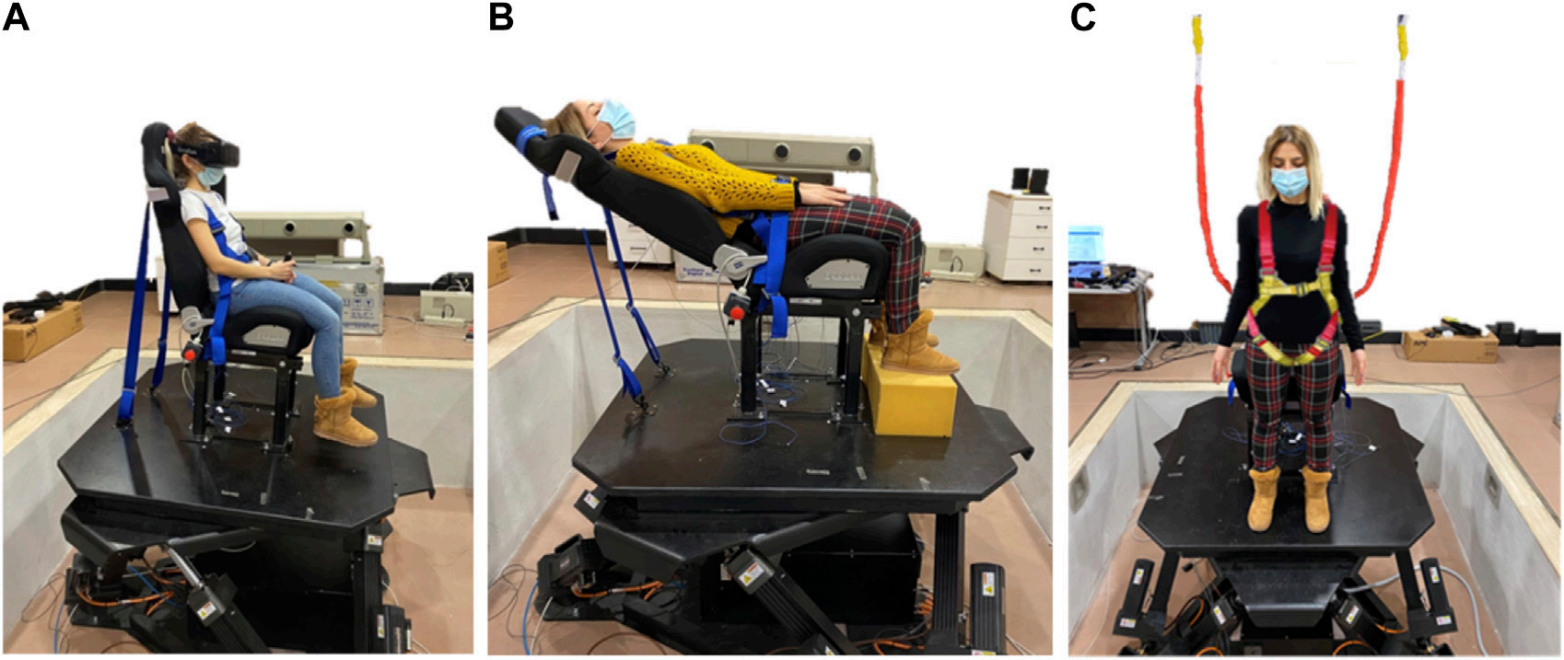
Different exemplary positions and/or orientations of the participant’s head relative to the motion platform. Participant seated straight in the chair **(A)**, seated reclined in the chair **(B)** or standing erect on the platform **(C)** with safety straps.

The adequate stimuli for naturalistic vestibular stimulation are represented by angular and linear accelerations of the head and static head tilt relative to gravity. In order to engage all vestibular sensors together or selectively, one must be able to move the person passively in all six degrees of freedom (DOF), by either translating linearly or rotating axially the head in 3-dimensional space. Stewart platforms are typically used for this purpose, since they generate these 6 DOFs movements with high temporal and spatial precision (e.g., Grabherr et al., 2008; MacNeilage et al., 2010; Crane, 2013; Ertl et al., 2022; La Scaleia et al., 2023). A Stewart platform consists of two rigid bodies (referred to as the base frame and the flying frame) connected through six extensible legs, each with spherical joints at both ends or with a spherical joint at one end and a universal joint at the other. The six extensible legs (hydraulic or electric linear actuators) are attached in pairs to three positions on the fixed base frame, crossing over to three mounting points below the flying frame (Dasgupta and Mruthyunjaya, 2000; Furqan et al., 2017). The person to be tested is placed above the flying frame, movable through 3D space in 6-DOF (Figure 1).

In line of principle, primary stimulation of the vestibular end organs might require the best possible alignment of the externally imposed accelerations (rotational and/or translational) according to the anatomical geometry of the semicircular canals and otoliths (Wagner et al., 2022). The direction of the stimuli is also considered critical when using and interpreting the video head impulse test (vHIT) in vestibular testing (Curthoys et al., 2023). However, the orientation of the vestibular sensors within the head varies considerably across individuals, and it is generally unknown unless one obtains high-resolution magnetic resonance imaging of the inner ear (Bögle et al., 2020; Wu et al., 2021). Moreover, ipsilateral semicircular canals are not strictly orthogonal between each other, and bilateral pairs of canals are not strictly parallel between each other (Wu et al., 2021). Also, saccules and utricles are not planar and contain diverse orientations of hair cells (Goldberg et al., 2012).

Critically, however, the vestibular apparatus is designed to respond to arbitrary directions of stimuli and report the instantaneous orientation and motion of the head. Therefore, in practice, the motion stimuli should be aligned as close as possible with the cardinal axes of the head (naso-occipital, interaural, cranio-caudal). A common solution consists in holding the participant’s head in a predefined place via a restraint, such as a helmet carefully centered relative to the axes of rotation. However, this procedure could be uncomfortable for some participants. It might also be inaccurate, with errors of ∼1 cm (Grabherr et al., 2008; Mallery et al., 2010; Valko et al., 2012), and head position and orientation may change during the experimental stimulation. Another critical issue is that a tight restraint (such as a helmet) may result in a satisfactory stabilization of head position, but also in unwanted somatosensory inputs. In fact, minimal head shifts within the restraint and/or contact forces arising at the head-restraint interface generate potentially strong somatosensory stimuli. If the stimulation procedure is aimed at testing vestibular motion perception, such somatosensory stimuli represent a confound, since they can provide significant motion cues adding on top of the vestibular cues (Chaudhuri et al., 2013).

Importantly, clinical applications of vestibular stimulation, such as those of quantitative assessment of motion perception deficits due to vestibulopathies or aging, often require the least stressful setup. Wearing a tight helmet may engender anxiety, due to claustrophobic or other emotionally negative feelings. Moreover, health-related restrictions on tight-fitting head-instruments, such as the restrictions due to the recent COVID-19 pandemic, may prohibit their use *tout court* (for instance, this was the case in our laboratory).

Finally, the need for monitoring head position and orientation in the absence of external constraints arises if the experiment involves a protocol in which participants can freely move their head, for instance to track a visual or auditory target, to explore the environment, etc. This would also be the case in the study of whole-body postural perturbations of standing subjects using a motion platform (see Figure 1C). In such case, since the head typically moves as a result of the perturbation, it would be important to monitor its motion and feed it back to update the motion of the platform and/or that of visual stimuli in real-time. Head-centric motion stimulations are also relevant in the context of human-machine integrations aimed at employing sensations that provide feedback to facilitate high bodily ownership and agency (Mueller et al., 2021).

To address these issues, we propose an approach to deliver platform motion stimuli aligned with head-fixed axes under head-free conditions. The problem of updating the motion profile of a platform based on the subject’s movements has also been investigated in a few previous studies. Thus, Huryn et al. (2010) and Luu et al. (2011) developed and validated algorithms to move a Stewart platform, programmed with the mechanics of an inverted pendulum, in order to control the movement of the body of a standing participant in response to a change in the applied ankle torque. However, in their algorithms, the position and orientation of the participants relative to the Stewart platform was fixed and the only tested motion profile was a rotation in pitch around the axis that passed through the ankle joints. Here, on the other hand, we propose a procedure to control a motion platform using a moving reference frame (i.e., head-fixed axes under head-free conditions). To the best of our knowledge, this is the first attempt to address this issue.

We document algorithms for the identification of head-fixed axes (H_RF_) using a motion capture system, and for the definition of an appropriate motion profile to perform translations and/or rotations of the H_RF_ with a motion platform. To accomplish the latter goal, the H_RF_ axes are mapped to the platform reference frame (S_RF_). We report the following steps: a) spatial calibration procedure to estimate the roto-translation matrix required to map the 3D data acquired in the motion capture reference frame (MC_RF_) into the platform reference frame (S_RF_), b) identification of head axes based on the Frankfurt plane in S_RF_, c) definition of a mobile reference frame H_RF_ with the three orthogonal axes of the head as coordinate axes, d) definition of the motion profile providing the required vestibular stimulation (rotation and/or translation relative to the H_RF_), e) remapping of the head motion profile in H_RF_ into a motion profile of the Stewart platform in S_RF,_ f) feasibility check of the motion profile to verify its compatibility with the platform physical limits, and g) real-time execution of the motion profile and head motion monitoring. We test the procedures in two different conditions: 1) the participant keeps the head in a resting position relative to the flying base, i. e., the head can assume a fully unrestrained posture with an arbitrary orientation relative to the flying base, while inter-aural, naso-occipital or cranio-caudal translations are applied, 2) the participant moves the head continuously while a naso-occipital translation is applied. The motion profile used to validate the procedure is a single cycle of sinusoidal acceleration, which is the typical motion profile used in protocols to evaluate vestibular motion perception (e.g., Benson et al., 1986; Valko et al., 2012; Bermúdez Rey et al., 2016; Bremova et al., 2016; Kobel et al., 2021a; La Scaleia et al., 2023).

## 2 System integration–hardware and software frame-work

### 2.1 The system

**FIGURE 2:**
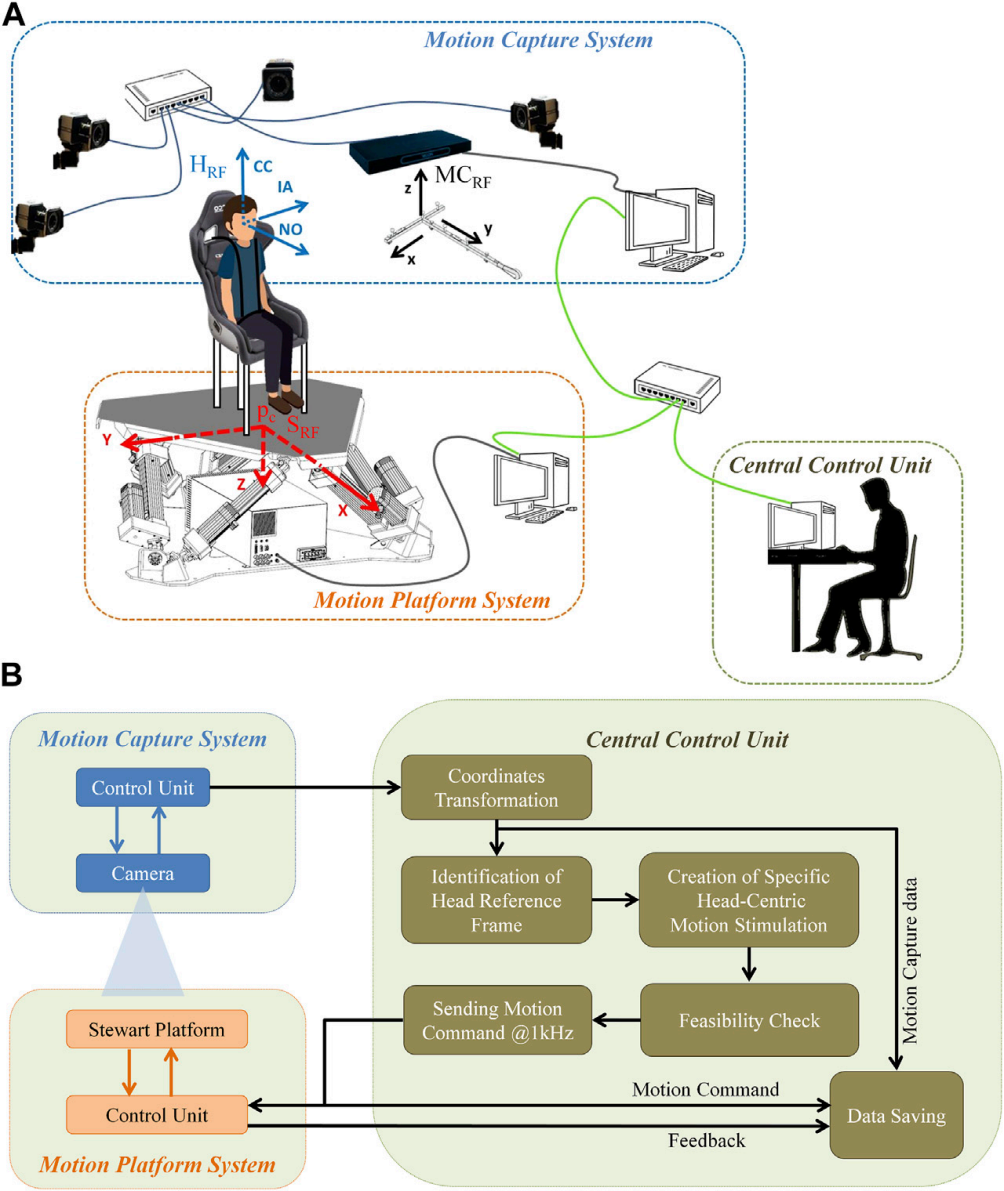
Schematic set-up. **(A)** The Central Command station integrates the motion capture system (e.g., Vicon) and the motion platform (e.g., MOOG MB-E-6DOF/12/1000). The different reference frames are shown for the participant’s head (H_RF_), the motion capture system centered in the calibration wand (MC_RF_), and the motion platform (S_RF_) in blue, black, and red respectively. **(B)** Schematic of the design of hardware interactions with the Central Control Unit. The Central Control Unit connects to the motion platform and to the motion capture system via UDP. The Central Control Unit is also in charge of determining the head reference frame, producing the specific head-centric motion stimuli, validating the movement’s feasibility and recording the entire communication into a log-file, which can be accessed for post-processing analysis.

The system includes a Stewart platform, a motion capture system, and a control server (Central Control Unit) that enables system integration and communication, as well as the validation of the feasibility of the desired movement. The schematic setup and the block diagram are illustrated in Figures 2A, B, respectively.

The six actuators of the Stewart platform are powered to modify their lengths, thereby changing the POSE (position and orientation) of the flying base in a controlled way. The origin of S_RF_ is the flying base motion centroid position when the flying base is in its home settled position. Motion centroid is the centroid of the joints below the flying base of the Stewart platform. When the flying base is at home all actuators are fully retracted (with a predefined tolerance) into the mechanical stops. *X-Y* plane is the plane that fits, in a least squares sense, the joints below the flying frame. *Z*-axis is orthogonal to the flying base and oriented toward the fixed base of the platform. The POSE of the platform corresponds to the position of the flying base centroid (***p***_***c***_) and the orientation of the flying base (Roll, Pitch, Yaw) in S_RF_ ( $p_{cx}^{S},p_{cy}^{S},p_{cz}^{S}$ , *Roll* ^*S*^, *Pitch* ^*S*^, *Yaw* ^*S*^), i.e., the POSE of the flying base is relative to initial POSE of the flying base when it is settled at home. The POSE is controlled (motion command) and monitored (feedback) by the hardware of the specific Stewart platform. The position and orientation of the head (H_RF_), monitored by the motion capture system in real-time in MC_RF_, must be mapped to a position and orientation in the flying base reference system ( $H_{\text{RF}}^{S}$ ). The platform POSE, which ensures the desired head motion profile, is generated starting from the measured $H_{\text{RF}}^{S}$ . Here we assume that the Stewart platform command and feedback are both expressed in the mode Degrees of Freedom (i.e., POSE).

### 2.2 Spatial calibration procedure

The spatial calibration procedure estimates the transformation between the motion capture reference frame (MC_RF_) and the platform reference frame (S_RF_), in order to map the 3D data acquired in MC_RF_ into S_RF_, i.e., it finds the relative rotation between the XYZ axes of the flying base reference system (S_RF_) and the XYZ axes of motion capture reference system (MC_RF_). The procedure also finds the relative position of the origin of MC_RF_ relative to the origin of S_RF_.

A typical spatial calibration procedure consists in finding the rigid Euclidean transformation that aligns two sets of corresponding 3D data collected in two different reference frames, i.e., computing the rotation matrix ( $R_{MC}^{S}$ ) and the translation vector ( $T_{MC}^{S}$ ) of the coordinate transformations between the two reference systems:

$$p_{i}^{S}=R_{MC}^{S}*p_{i}^{MC}+T_{MC}^{S}+N_{i}$$ (1)

where ***p***_*i*_^*S*^ and ***p***_*i*_^*MC*^ are the position vectors at time t_i_ of a point with respect to the origin of the reference frame S_RF_ and MC_RF,_ respectively, ***N***_***i***_ is the noise vector that accounts for measurement noise; $p_{i}^{S}$ , $p_{i}^{MC},T_{MC}^{S},N_{i}$ are 3 × 1 vectors, $R_{MC}^{S}$ is a 3 × 3 rotation matrix, the symbols ‘*’ denotes the product between a matrix and a vector. A critical issue here is that no knowledge of the 3D coordinates of m points in the two reference systems S_RF_ and MC_RF_ is available. Indeed, Stewart platforms typically provide real-time feedback only about the actual POSE of the flying base relative to the reference system S_RF_. Alternatively, the platforms can provide the length of the six actuators relative to their initial length when the flying base is settled at home. The flying base motion centroid is the centroid of the joints below the flying base of the Stewart platform, therefore it can be considered a virtual point in air. It is not possible to place motion capture markers on the moving joints below the flying base of the Stewart platforms and then compute the centroid position of the flying base in MC_RF_. Moreover, it is not possible to place a marker on the motion centroid in air or to place markers in the six actuators’ axes. Furthermore, even when the platform centroid is accessible, the manual positioning of a marker could be inaccurate, increasing the noise in $R_{MC}^{S}$ and $T_{MC}^{S}$ estimation.

To overcome the above-mentioned critical issues, we take advantage of the platform motion to estimate $R_{MC}^{S}$ and $T_{MC}^{S}$ .We use two different sets of platform movements: pure translation movement to estimate $R_{MC}^{S}$ and pure rotation movement to estimate $T_{MC}^{S}$ (for details see Sections 2.2.1, 2.2.2).

The predefined motion profile (pure translation or rotation) sampled at 1 kHz is sent to the motion control system of the Stewart platform as an ordered sequence of point-to-point positions of the flying base’ motion centroid. The actual positions of this motion centroid (***p***_c_) are recorded in S_RF_ using the feedback POSE from the platform (the actual positions of the motion centroid versus time, sampled at 1 kHz, are estimated from the feedback received from the encoders built into the 6 parallel actuators and they are collected by the Moog Control Unit, see Figure 2B), and can be expressed in MC_RF_ by means of a motion capture system that acquires the position of one or more markers strictly fixed to the motion platform (***p***_Marker(s)_). Markers can be fixed anywhere on the platform as long as they are always visible to the motion capture system during both sets of platform movements and they are one with the platform during its movement.

Using the platform motion, we can collect more than one point with the platform feedback ( ${p_{c}}_{i}$ ) and with motion capture system for each marker ( ${p_{Marker\left(s\right)}}_{i}$ ) (the POSE varies during the movement and ‘i’ is the sample collected at time t_i_ during the movement). 3D position data of the flying base centroid ( $p_{c}^{S}$ ) and of the markers ( ${p_{Marker}}^{MC}$ ) attached to the platform are collected by the Central Control Unit (Figure 2B). We need to ensure that the 3D data (obtained from both platform feedback and motion capture system), even if they are related to distinct physical points ( $p_{c}$ and $p_{Marker\left(s\right)}$ ), are paired data, i.e., they correspond to the same POSE of the platform at the same time t_i_. The 3D position data are collected from the time the start movement command is sent to the platform, when the platform is motionless in the starting position, and over the entire duration of the motion profile execution. Neither the sharing of a trigger signal or the sharing of a common clock guarantees the synchronous acquisition of points in the Moog and motion capture system. Indeed, the latency of Moog motion, Moog motion recording and motion capture recording after the start command are unknown. Moog latency may vary depending on the movement profile commanded, the motion capture latency may vary depending on the number of acquired markers, the acquisition frequency and the number of cameras used. Moreover 3D data provided by the Stewart platform and by the motion capture system can be sampled at different frequencies, depending on the specific systems used. In case of different acquisition rates of the platform feedback and of the motion capture system, the data sampled with the lower frequency must be interpolated at the frequency of the data sampled at the higher acquisition rate. Given the possible temporal asynchrony between the data provided by the platform feedback and the ones provided by the motion capture system, we use 5 cycles of sinusoidal motion and compute the phase difference between the signals recorded by the two systems. The temporal alignment of the signals can be obtained in various ways, e.g., using cross-correlation analysis or evaluating the difference between the phases of the sinusoids fitted to the two datasets. Here, we propose to use a subset of data (4 complete cycles) starting from the first peak of the sinewave for each recorded signal in order to obtain the paired data, from the two systems (platform feedback and motion capture system), corresponding to the same POSE of the platform. The procedure is described in the Supplementary Appendix A1, paragraph S.1.

#### 2.2.1 Evaluation of rotation matrix

The rotation matrix is calculated from the motion data acquired during the execution of pure translation movements of the platform. In this manner, since the flying base orientation is fixed during the movement, the traveled distance by the centroid and by the markers is the same even if the 3D points are distinct physical points. The relative motion of the flying motion base with respect to its initial position and orientation at the beginning of the motion (in the reference system S_RF_) and the relative motion of the markers fixed to the Moog with respect to their initial positions (in the reference system MC_RF_) can be used to estimate the relative rotation between the axes of the two reference systems S_RF_ and MC_RF_.

In order to minimize errors in the rotation matrix estimation, the 3D points collected in S_RF_ ( $p_{c}$ ) and MC_RF_ ( $p_{Marker\left(s\right)}$ ) must not lie on a plane and must cover a significant portion of the volume that the platform can explore. Furthermore, it must be ensured that points obtained from both platform feedback ( ${p_{c}}_{i}$ ) and motion capture system ( ${p_{Marker\left(s\right)}}_{i}$ ) correspond to the same POSE (*i*) of the motion platform. The correct coupling of the measurements obtained by the platform feedback and motion capture system could be guaranteed, regardless of the acquisition frequencies and any acquisition asynchronies, by using multiple static positions (with the same orientation) of the platform that explore the entire workspace. Nevertheless, acquiring a large number of 3D points using multiple stationary platform positions would take a long time.

To reduce the duration of the calibration procedure, here we propose to use one pure translation movements of the platform for each axis of S_RF_ (antero-posterior, lateral, and vertical). We use three orthogonal translations to guarantee that the collected points are not contained in a plane, and cannot be fitted in a robust way by a plane. A single trial consists of 5 consecutive cycles of sinusoidal motion along x^S^, y^S^, and z^S^ in order to have multiple measure of the same spatial points. The sinusoidal motion profile has zero phase starting from a predefined initial position (IP) of the flying base centroid to ensure that the oscillation is centered on the IP. The IP around which the flying base centroid oscillates can be in any arbitrary point of the workspace of the flying base centroid. We choose the neutral position at the center of the workspace of the flying base centroid as the IP to have a greater volume to explore during the calibration movements. To ensure accurate capture of the executed motion profile the sinusoidal motion frequency is 0.1 Hz, which is significantly lower than the acquisition frequencies of the motion platform and motion capture system, the amplitude is 0.1 m, in order to explore a significant portion of the flying base centroid’s workspace while also ensuring that the speed and the acceleration of execution of the movement is not excessively high, and the markers must be always visible to the motion capture system during the entire motion.

For each trial, the 3D position data of the flying base centroid ( $p_{c}^{S}$ ) and of the markers ( $p_{Marker}$ ^*MC*^) attached to the platform are collected. Then, the platform returns home at the end of the motion profile execution (at the end of each trial). The 3D position data are collected from the time the start movement command is sent to the platform, when the platform is motionless in IP, and over a time epoch of 50 s, i.e., the duration of each trial (5 cycles x 10 s duration of a single cycle).

We must ensure that the collected data (obtained from both platform feedback and motion capture system) start with the coordinates of the platform when it is motionless in IP. The data sampled with the lower frequency are interpolated at the frequency of the data sampled at the higher acquisition rate. The 3D data are then time-aligned (a possible approach is in Supplementary Appendix A1, paragraph S.1) in order to have the paired-3D data ( ${{p_{Marker}}_{i}}^{MC}$ and ${{p_{c}}_{i}}^{S}$ ) for the *i*th POSE of the platform.

In order to find the transformation between the motion capture reference frame (MC_RF_) and the platform reference frame (S_RF_), i.e., in order to map the 3D data acquired in MC_RF_ into S_RF_, we first estimate the relative rotation between the XYZ axes of motion capture reference system (MC_RF_) and the XYZ axes of the flying base reference system (S_RF_) as follows.

The position of the platform centroid in MC_RF_ for each *i*th sample can be expressed as:

$${{p_{c}}_{i}}^{MC}={{p_{Marker}}_{i}}^{MC}+L_{Marker}^{MC}$$ (2)

where $L_{Marker}^{MC}$ represents the 3 × 1 (unknown) translation vector, in MC_RF_, that translates the position of a given marker ( ${p_{Marker}}^{MC}$ ) to the position of flying base centroid ( $p_{c}^{MC}$ ).

Since we use pure translation movements, for a given marker, $L_{Marker}^{MC}$ is fixed for each *i*th sample, so the least square solutions of Eq. 1 for $R_{MC}^{S}$ and $T_{MC}^{S}$ ( $\left.\hat{R_{MC}^{S}},\hat{T_{MC}^{S}}\right)$ are given by:

$$\left(\hat{R_{MC}^{S}},\hat{T_{MC}^{S}}\right)=\underset{R_{MC}^{S},T_{MC}^{S}}{\mathrm{argmin}}\sum_{i=1}^{m}{\left\|\left(R_{MC}^{S}*{{p_{c}}_{i}}^{MC}+T_{MC}^{S}\right)-{{p_{c}}_{i}}^{S}\right\|}^{2}=\underset{R_{MC}^{S},T_{MC}^{S}}{\mathrm{argmin}}\sum_{i=1}^{m}{\left\|\left(R_{MC}^{S}*\left({p_{Marker}}_{i}^{MC}+L_{Marker}^{MC}\right)+T_{MC}^{S}\right)-{{p_{c}}_{i}}^{S}\right\|}^{2}$$ (3)

where $\hat{R_{MC}^{S}}$ and $\hat{T_{MC}^{S}}$ are maximum likelihood estimates of $R_{MC}^{S}$ and $T_{MC}^{S}$ , m is the number of non-aligned 3D points and ${{p_{Marker}}_{i}}^{MC}$ and ${{p_{c}}_{i}}^{S}$ are the paired positions of the two distinct points, marker and flying base centroid, with the platform in a given POSE in the *i*th sample.

The least squares problem (Eq. 3) can be simplified by finding the centroids of both datasets ( ${P_{c}}^{s}$ for platform feedback data and ${P_{Marker}}^{s}$ for the recorded marker in MC_RF_) and bringing both datasets at the origin:

$${p_{detrend}}_{i}^{MC}={p_{Marker}}_{i}^{MC}-{P_{Marker}}^{MC};{p_{detrend}}_{i}^{S}={p_{c}}_{i}^{S}-{P_{c}}^{s};$$ (4)

If more than one marker is collected by the motion capture system, the mean position of the recorded markers, for each *i*th point, is assessed before the evaluation of ${p_{detrend}}_{i}^{MC}$ .

We can solve Eq. 3 by finding the maximum likelihood estimate of the rotation matrix $\hat{R_{MC}^{S}}$ by means of Eqs 2–4:

$$\begin{array}{ll} \hat{R_{MC}^{S}} & =\underset{R_{MC}^{S}}{\mathrm{argmin}}\sum_{i=1}^{m}{\left\|{\left(R\right.}_{MC}^{S}*\left({p_{detrend}}_{i}^{MC}+{P_{Marker}}^{MC}+L_{Marker}^{MC}\right)+T_{MC}^{S})-{P_{c}}^{s}-{p_{detrend}}_{i}^{S}\right\|}^{2}\\ & =\underset{R_{MC}^{S}}{\mathrm{argmin}}\sum_{i=1}^{m}{\left\|R_{MC}^{S}*{p_{detrend}}_{i}^{MC}+\left(R_{MC}^{S}*\left({P_{Marker}}^{MC}+L_{Marker}^{MC}\right)+T_{MC}^{S}\right)-{P_{c}}^{s}-{p_{detrend}}_{i}^{S}\right\|}^{2}\\ & =\underset{R_{MC}^{S}}{\mathrm{argmin}}\sum_{i=1}^{m}{\left\|R_{MC}^{S}*{p_{detrend}}_{i}^{MC}-{p_{detrend}}_{i}^{S}\right\|}^{2} \end{array}$$ (5)

Several efficient methods have been developed to compute $\hat{R_{MC}^{S}}$ as the solution to a least squares formulation (Eq. 5). We suggest using closed form solutions because they are generally more efficient and robust than iterative methods. The latter may suffer from the problems of not guaranteeing convergence, becoming trapped in local minima of the error function, and requiring a good starting estimate. The various approaches based on the closed form solutions differ in terms of the representation used for the transformation, and the mathematical derivation of the solution (see Eggert et al., 1997; Sarabandi and Tomas, 2023).

One possible approach to solve Eq. 5 was developed by Arun et al., 1987. It is based on computing the singular value decomposition (SVD) of a covariance matrix, obtained from the matrix product between the two datasets after moving the origin of the two coordinate systems to the point set centroids. Another related approach, but based on exploiting the orthonormal properties of the rotation matrix, computes the eigensystem of a different derived matrix (Horn et al., 1988). Another algorithm, also developed by Horn (1987), involves computing the eigensystem of a matrix related to the representation of the rotational component as a unit quaternion.

Here we summarize the method developed by Arun et al., 1987.

Eq. 5 can be written as:

$$\hat{R_{MC}^{S}}=\underset{R_{MC}^{s}}{\mathrm{argmin}}\sum_{i=1}^{m}\left({\left({p_{detrend}}_{i}^{MC}\right)}^{T}*{p_{detrend}}_{i}^{MC}+{\left({p_{detrend}}_{i}^{S}\right)}^{T}*{p_{detrend}}_{i}^{S}-2*{\left({p_{detrend}}_{i}^{S}\right)}^{T}*R_{MC}^{S}*{p_{detrend}}_{i}^{MC}\right)$$ (6)

Eq. 6 is minimized when the last term is maximized:

$$\begin{array}{l} \hat{R_{MC}^{S}}=\underset{R_{MC}^{s}}{\mathrm{argmax}}\left(\sum_{i=1}^{m}{\left({p_{detrend}}_{i}^{S}\right)}^{T}*R_{MC}^{S}*{p_{detrend}}_{i}^{MC}\right)\\ \underset{R_{MC}^{s}}{=\mathrm{argmax}}\left(\text{Trace}\left({\left({p_{detrend}}^{S}\right)}^{T}*R_{MC}^{S}*{p_{detrend}}^{MC}\right)\right)\\ \underset{R_{MC}^{s}}{=\mathrm{argmax}}\left(\text{Trace}\left({p_{detrend}}^{MC}*{\left({p_{detrend}}^{S}\right)}^{T}*R_{MC}^{S}\right)\right) \end{array}$$ (7)

where ***p***_*detrend*_ is the 3×m matrix with ${p_{detrend}}_{i}$ as its columns, and we take advantage of the property that Trace(A*B) = Trace(B*A). Therefore, the best-fitting rotation $\hat{R_{MC}^{S}}$ can be found by applying the Singular Value Decomposition to the covariance matrix ***H***, obtained from the matrix product between the two datasets:

$$H={p_{detrend}}^{MC}*{\left({p_{detrend}}^{S}\right)}^{T}$$ (8)

$$H=U*S{*V}^{T}$$ (9)

where the columns of ***U*** and ***V*** (orthogonal matrices) consist of the left and right singular vectors, respectively, and ***S*** is a 3 × 3 diagonal matrix, with non-negative elements, whose diagonal entries are represented by the singular values of ***H***.

By substituting the decomposition into the trace that we have to maximize, we obtain:

$$\text{Trace}\left(U*S*V^{T}*R_{MC}^{S}\right)=\text{Trace}\left(R_{MC}^{S}*U*S{*V}^{T}\right)=\text{Trace}\left(S*V^{T}*R_{MC}^{S}*U\right)$$ (10)

Since ***S*** is a diagonal matrix with non-negative values, and ***V***, $R_{MC}^{S}$ and ***U*** are all orthogonal matrices ( $V^{T}*R_{MC}^{S}*U$ is also an orthogonal matrix), the trace is maximized when $V^{T}*R_{MC}^{S}*U=I$ , an identity matrix. Accordingly,

$$\hat{R_{MC}^{S}}={V*U}^{T}$$ (11)

Of course, the larger is the dataset of non-aligned points (m), the better is the estimation of ***H***, and therefore the smaller is the error resulting from the minimization procedure in the rotation matrix computation.

#### 2.2.2 Evaluation of translation vector

The translation vector $T_{MC}^{S}$ between S_RF_ and MC_RF_ is calculated from the motion data acquired during the execution of pure rotational movements of the platform. In this manner, the flying base centroid (***p***_***c***_) is fixed during the movement and, since none of the markers are placed on the rotation centroid of the platform (the platform centroid is a virtual non-physical point), the trajectory travelled by each marker lies on a specific marker-sphere, whose radius corresponds to the distance between the marker and ***p***_***c***_ (the center of each marker-sphere).

We propose to use five cycles of sinusoidal rotation with frequency 0.1 Hz and amplitude 10° for Roll^S^, Pitch^S^, and Yaw^S^ rotations around $p_{c}^{S}$ . The amplitude and the frequency of the rotations can be chosen arbitrarily, the only constraints are that the whole rotation motion (e.g., ±10°) around the initial orientation axes of the flying base is feasible, the trajectories of the markers attached to the platform during the three rotational movements are not contained within a single plane, the markers are always visible to the motion capture system during the entire motion, and the motion frequency is much lower than the acquisition frequencies of the motion platform and motion capture system.

With these motion profiles, each marker moves along arcs of circle that lie on a specific marker-sphere, whose radius corresponds to the distance between the marker and ***p***_***c***_. When more than one marker is used for this purpose, the resulting spheres have different radii but the same center. This center corresponds to ***p***_***c***_.

With a least squares sphere fitting, we calculate the center [*x*_*cj*_^*MC*^, *y*_*cj*_^*MC*^, *z*_*cj*_^*MC*^]^T^ of the sphere over which the marker *j* has moved during the rotational movements (Jennings, 2022). For a given set of m markers, we have m estimates of the position of the flying base motion centroid in the motion capture reference system MC_RF_. ${p_{c}}^{MC}$ can be estimated as the average position of the m sphere centers in the motion capture reference system, with the following equation:

$${p_{c}}^{MC}=\left(\begin{array}{c} x_{c}^{MC}\\ \begin{array}{c} y_{c}^{MC}\\ z_{c}^{MC} \end{array} \end{array}\right)=\left(\begin{array}{c} \sum_{j=1}^{m}\frac{x_{cj}^{MC}}{m}\\ \sum_{j=1}^{m}\frac{y_{cj}^{MC}}{m}\\ \sum_{j=1}^{m}\frac{z_{cj}^{MC}}{m} \end{array}\right)$$ (12)

where *x*_*cj*_^*MC*^, *y*_*cj*_^*MC*^ and *z*_*cj*_^*MC*^ are the spatial coordinates in the MC_RF_ of the center of the sphere over which the marker *j* has moved, and m is the number of recorded markers.

The best-fitting translation vector $\hat{T_{MC}^{S}}$ , according with Eqs 1, 3, is obtained as the distance between the two corresponding points ( $p_{c}^{S}$ and ${p_{c}}^{MC}$ ), applying the rotation matrix $\hat{R_{MC}^{S}}$ :

$$\hat{T_{MC}^{S}}=p_{c}^{S}-\hat{R_{MC}^{S}}{{*p}_{c}}^{MC}$$ (13)

## 3 Identification of head-fixed axes

### 3.1 Frankfurt plane of the head

**FIGURE 3:**
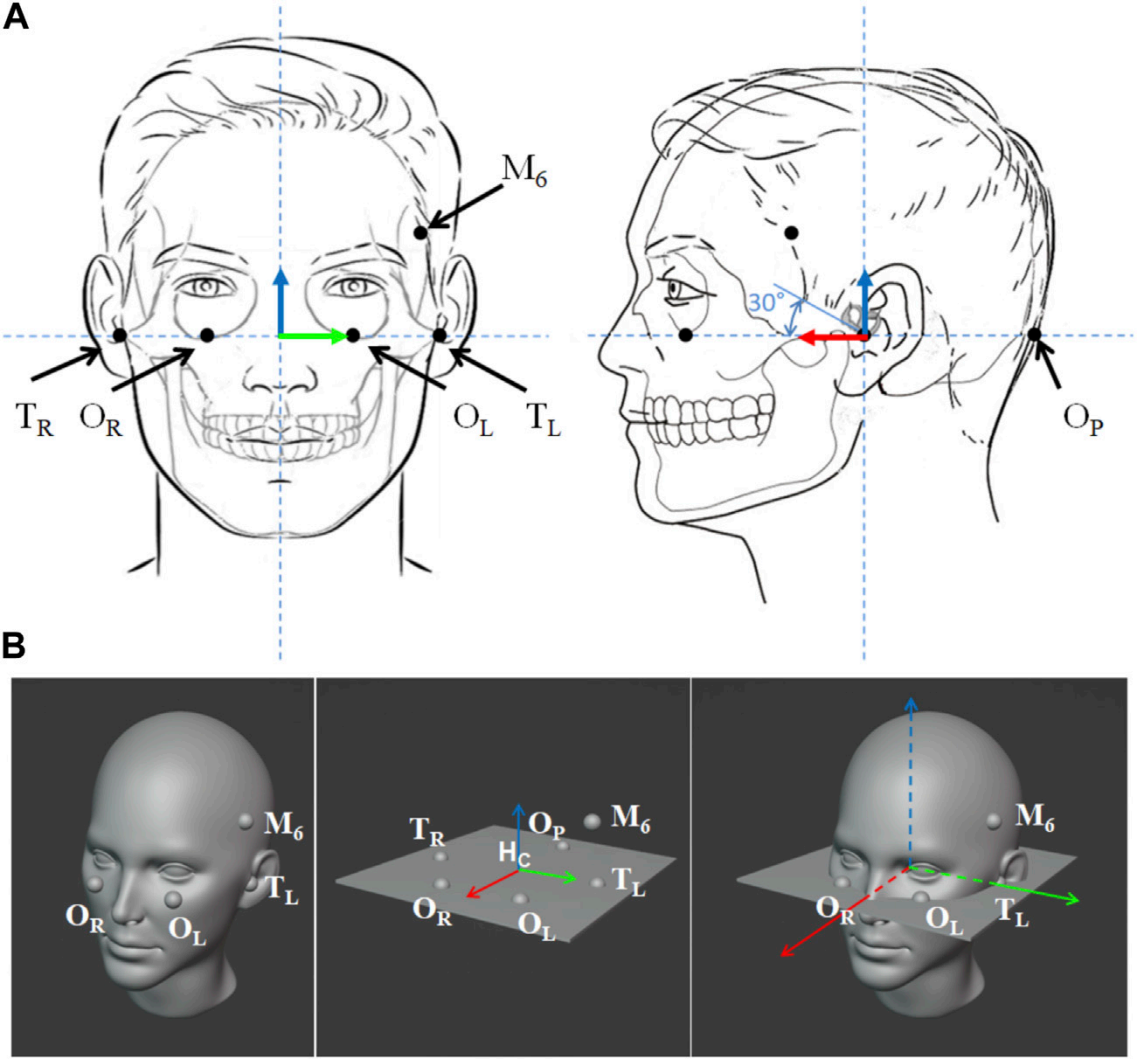
Markers placement to identify head position and orientation. **(A)** The left and right orbitales (O_L_, O_R_) are the lowest points of the lower margin of the left and right orbits (eye sockets). The porions correspond to the left and right superior margins of tragus (T_L_, T_R_), the most lateral points of the roofs of the left and right ear canals. Near the middle of the squamous part of the occipital bone is the external occipital protuberance, occiput (O_P_). The marker M_6_ is placed in an asymmetric position compared to the other markers and it is used to individuate the orientation of the cranio-caudal axis. Blue, red and green arrows represent the naso-occipital, inter-aural and cranio-caudal axes reconstructed with the aforementioned markers. The lateral semicircular canal is typically oriented at about 30° from the horizontal plane (FP). **(B)** The Frankfurt plane (FP) is defined as a plane that passes through the orbitales and the porions. FP is identified using the least square method of the normal distance of $O_{L}^{\text{MC}}$ , $O_{R}^{\text{MC}}$ , $T_{L}^{\text{MC}},T_{R}^{\text{MC}}$ , and $O_{P}^{\text{MC}}$ to the plane. The center of the head (H_c_) lies on FP. The naso-occipital, inter-aural and cranio-caudal axes in the same format of **(A)**.

The anatomical coordinate system of the head can be based on the Frankfurt plane (FP), i.e., the plane through the inferior margin of the left orbit (O_L_) and the external auditory meatus of both ears (T_L_, T_R_). FP is often used to orient the head so that the plane is horizontal in standardized exams (Moorrees and Kean, 1958). To identify the Frankfurt plane in each experimental subject, we place 5 markers on the head over the following anatomical landmarks (see Figure 3): left orbital inferior margin (O_L_), right tragus (T_R_), left tragus (T_L_), right orbital inferior margin (O_R_), occiput (O_P_). An additional marker not lying in the FP plane is placed in an arbitrary position on the forehead (M_6_). This additional marker is placed on the head in an asymmetric position compared to the other markers, and it is used to identify the orientation of the cranio-caudal axis. To reduce the effect of measurement noise, the FP is identified using the least square method of the normal distance of 5 markers to the plane. The motion capture system is used to record the position of the 5 markers ( $O_{L}^{\text{MC}}$ , $O_{R}^{\text{MC}}$ , $T_{L}^{\text{MC}},T_{R}^{\text{MC}}$ , and $O_{P}^{\text{MC}}$ ), which are then mapped into S_RF_ ( $O_{L}^{S}$ , $O_{R}^{S}$ , $T_{L}^{S},T_{R}^{S}$ , and $O_{P}^{S}$ ), using the above matrices ( $\hat{R_{MC}^{S}}$ and $\hat{T_{MC}^{S}}$ ).

The general form of the equation of a plane is:

ax+by+cz+d=0 (14)

where a, b, c are the Cartesian components of the unit normal vector ***n*** = [a b c]^T^. The column vector ***n*** is parallel to the cranio-caudal axis of the head (***CC***). Estimates of the unknown coefficients a, b, c are determined by minimizing the sum of squared orthogonal distances between the points ***q***_*i*_^*S*^ ( $O_{L}^{S}$ , $O_{R}^{S}$ , $T_{L}^{S},T_{R}^{S}$ , and $O_{P}^{S}$ ) and the plane.

A possible procedure to identify FP and ***n*** is detailed below.

We must specify *a priori* a single point on the plane, and a plausible choice is given by the centroid of all measures, denoted as ***C*** ^*S*^. Assuming that measurement errors (for example, due to inaccurate positioning of the markers) are random and unbiased, the plane should pass through the centroid of the measurements.

Accordingly, we define:

$$C^{S}=\left(\begin{array}{c} x_{C}^{S}\\ \begin{array}{c} y_{C}^{S}\\ z_{C}^{S} \end{array} \end{array}\right)=\left(\begin{array}{c} \sum_{i=1}^{k}\frac{x_{i}^{S}}{k}\\ \sum_{i=1}^{k}\frac{y_{i}^{S}}{k}\\ \sum_{i=1}^{k}\frac{z_{i}^{S}}{k} \end{array}\right)$$ (15)

where *x* ^*S*^,*y* ^*S*^ and *z* ^*S*^ are the spatial coordinates in S_RF_, and k = 5 is the number of measured points.

Let v be the vector from ***C*** ^*S*^ to ***q***_*i*_^*S*^ = [*x*_*i*_^*S*^ *y*_*i*_^*S*^ *z*_*i*_^*S*^]^T^

$$v_{i}={\left[{x_{i}}^{S}-{x_{C}}^{S},{y_{i}}^{S}-{y_{C}}^{S},{z_{i}}^{S}-{z_{C}}^{S}\right]}^{T}$$ (16)

The distance Dist_i_ of ***q***_*i*_^*S*^ to the plane is equal to the length of the projection of v over the vector ***n***:

$${\text{Dist}}_{i}=\left|v_{i}∙n\right|=\frac{a*\left({x_{i}}^{S}-{x_{C}}^{S}\right)+b*\left({y_{i}}^{S}-{y_{C}}^{S}\right)+c*\left({z_{i}}^{S}-{z_{C}}^{S}\right)}{\sqrt{a^{2}+b^{2}+c^{2}}}$$ (17)

In order to identify the FP, we have to find the vector ***n*** = (a, b, c)^T^ that minimizes:

$$\sum_{i=1}^{k}{\left({\text{Dist}}_{i}\right)}^{2}=\sum_{i=1}^{k}\frac{{\left[a*\right.\left({x_{i}}^{S}-{x_{C}}^{S}\right)+b*\left({y_{i}}^{S}-{y_{C}}^{S}\right)+c*\left.\left({z_{i}}^{S}-{z_{C}}^{S}\right)\right]}^{2}}{{\left(\sqrt{a^{2}+b^{2}+c^{2}}\right)}^{2}}$$ (18)

Given the matrix ***V*** and k markers

$$V=\left[\begin{array}{c} v_{1}^{T}\\ \vdots \\ v_{k}^{T} \end{array}\right]=\left[\begin{array}{ccc} \left(x_{1}^{S}-x_{C}^{S}\right) & \left(y_{1}^{S}-y_{C}^{S}\right) & \left(z_{1}^{S}-z_{C}^{S}\right)\\ \vdots & \vdots & \vdots \\ \left(x_{k}^{S}-x_{C}^{S}\right) & \left(y_{k}^{S}-y_{C}^{S}\right) & \left(z_{k}^{S}-z_{C}^{S}\right) \end{array}\right]$$ (19)

We can express Eq. 18 in matrix form

$$\sum_{i=1}^{k}\left({{\text{Dist}}_{i}}^{2}\right)=\frac{{\left(V*n\right)}^{T}*\left(V*n\right)}{n^{T}*n}=\frac{{n^{T}*V}^{T}*V*n}{n^{T}*n}=\frac{n^{T}*A*n}{n^{T}*n}$$ (20)

with ***A*** = ***V*** ^*T*^ * ***V***. Eq. 20 has the form of a Rayleigh quotient (Strang, 1988). Thus, the vector ***n*** that minimizes Eq. 20 is the minimum eigenvector (i.e., the eigenvector corresponding to the minimum eigenvalue) of matrix ***A***.

### 3.2 Head-fixed axes

We use a right-handed reference system to describe the head-fixed axes, naso-occipital (***NO***), inter-aural (***IA***) and cranio-caudal (***CC***), positive to the front, left ear, and upwards, respectively (see Figure 3). We first identify the inter-aural axis passing through the right and left tragus. To this end, we evaluate the orthogonal projection of both markers on the left and right tragus [***T***_*i*_^*S*^ (*x*_*i*_^*S*^; *y*_*i*_^*S*^; *z*_*i*_^*S*^), with *i* = 1, 2 corresponding to marker ***T***_*L*_ and ***T***_*R*_ in S_RF_ (Figure 3B)] on the FP.

Using the previously calculated point ***C*** ^***S***^ and the normal vector orthogonal to FP (***n***), the projection of ***T***_*i*_^*S*^ on FP plane is calculated as:

$$T_{i_{proj}}^{S}=T_{i}^{S}-\left(\left(T_{i}^{S}-C^{S}\right)∙n\right)*n$$ (21)

The inter-aural axis (***IA***) is defined as:

$$IA=\left(T_{L_{proj_{x}}}^{S}-T_{R_{proj_{x}}}^{S};T_{L_{proj_{y}}}^{S}-T_{R_{proj_{y}}}^{S};T_{L_{proj_{z}}}^{S}-T_{R_{proj_{z}}}^{S}\right)=\left({dx}_{IA};{dy}_{IA};{dz}_{IA}\right)$$ (22)

which has a unit vector:

$$u_{IA}^{s}=\frac{IA}{\left|IA\right|}=\frac{IA}{\sqrt{{dx}_{IA}^{2}+{dy}_{IA}^{2}+{dz}_{IA}^{2}}}$$ (23)

The center of the head (***H***_*c*_) is the midpoint of the line segment defined by $T_{L_{proj}}^{S}$ and $T_{R_{proj}}^{S}$ .

The orientation of the cranio-caudal axis ***CC*** (see Figure 3) can be obtained by finding the orientation of the vector $\vec{F}$ orthogonal to **FP** that point to the forehead marker $M_{6}^{S}$ . ***CC*** is the vector passing through ***H***_***c***_, normal to the FP and with the same orientation of vector $\vec{F}$ (i.e., the dot product of **CC** and **F** is positive):

$$u_{CC}^{s}=n\text{ with}\left(M_{6}^{s}-H_{c}^{s}\right)\cdot n>0;$$ (24a)

or

$$u_{CC}^{s}=-n\text{ with}\left(M_{6}^{s}-H_{c}^{s}\right)\cdot n<0$$ (24b)

The naso-occipital axis (***NO***) lies on the FP, is orthogonal to the inter-aural axis and cranio-caudal axis, and passes through the ***H***_***c***_:

$$u_{NO}^{s}=u_{IA}^{s}\times u_{CC}^{s}$$ (25)

## 4 Head-centric vestibular stimulation

In theory, the assessment of the vestibular function may require the alignment of the motion stimuli with the anatomic orientation of the sensory receptors in the inner ear. However, in practice this is often not feasible due to the significant inter-individual variability of anatomy of the vestibular apparatus and the lack of high-resolution imaging of the inner ear (see Introduction). Therefore, the simplest solution is to align the motion stimuli with the cardinal axes of the head (see Section 3.2), by taking advantage of the fact that the vestibular apparatus is designed to respond to arbitrary directions of stimuli and report the instantaneous orientation and motion of the head. In our case, the motion platform is translated along (or rotated about) the head axis ***u*** (interaural, naso-occipital, or cranio-caudal), with *u_x_* ^2^ + *u_y_* ^2^ + *u_z_* ^2^ = 1, which passes through ***H***_*c*_. POSE(*t*) = [ $p_{cx}^{S}$ *(t)*, $p_{cy}^{S}$ *(t)*, $p_{cz}^{S}$ *(t)*, *Roll* ^*S*^ *(t)*, *Pitch* ^*S*^ *(t)*, *Yaw* ^*S*^ *(t)*] represents the platform position and orientation ensuring the desired head-centric stimulus.

### 4.1 Translations

Here we translate the participant’s head along the selected head axis ***u*** = (*u_x_*, *u_y_*, *u_z_*) with a motion profile *s*(*t*).

We define the incremental displacement d*s*(*t*_*i*_) as:

$$ds\left(t_{i}\right)=v\left(t_{i}\right)*dt$$ (26)

with *t* = [0:d*t*:*t*_*end*_] (*t*_*end*_ is the total duration in seconds of the motion profile, and d*t* = 1/freq_command, with ‘freq_command’ equal to the control frequency of the motion platform), v (*t*_*i*_) is the instantaneous velocity at *t*_*i*_ and d*s*(*t*_*i*_) is the desired displacement in d*t* = *t*_*i+1*_ - *t*_*i*_.

The algorithm requires that, at the beginning of stimulation (*t* = 0), the position and orientation of the platform is determined (POSE(*t* = 0) = [ $p_{c0x}^{S}$ ; $p_{c0y}^{S}$ ; $p_{c0z}^{S}$ ; $Roll_{0}^{S}$ ; ${Pitch}_{0}^{s}$ ; $Yaw_{0}^{S}$ ]). The head orientation (***u*** (*t* = 0)) is defined within that motion platform configuration and monitoring during the stimulation (***u*** *(t)*). Next, the movement translation is applied along the selected head axis at the motion platform centroid (***p***_*c*_^*S*^(*t*)).

$$p_{c}^{S}\left(t_{i+1}\right)=p_{c}^{S}\left(t_{i}\right)+ds\left(t_{i}\right)*\left(u_{x}^{S}\left(t_{i}\right),u_{y}^{S}\left(t_{i}\right),u_{z}^{S}\left(t_{i}\right)\right)$$ (27)

The configuration of the platform that guarantees a translation of *s*(*t*) along the axis ***u*** passing through the ***H***_***c***_ is given by:

$$\text{POSE}\left(t_{i+1}\right)=\left[p_{c}^{S}\left(t_{i}\right)+ds\left(t_{i}\right)*\left(u_{x}^{S}\left(t_{i}\right),u_{y}^{S}\left(t_{i}\right),u_{z}^{S}\left(t_{i}\right)\right),{Roll}_{0}^{S},{Pitch}_{0}^{S},{Yaw}_{0}^{S}\right]$$ (28)

### 4.2 Rotations

Here we rotate the participant’s head by the angle *θ*(*t*) around the head axis ***u*** passing through ***H***_*c*_. As before, the algorithm requires identifying the POSE of the motion platform at the start of the stimulation, and then defining the head orientation (***u*** (*t = 0*)) and position (***H***_*c*_(*t = 0*)) within that motion platform configuration and during the stimulation (***u*** (*t*), ***H***_*c*_(*t*)).

Next, the incremental movement rotation d*θ*(*t*_*i*_), defined as the desired rotation in d*t* = *t*_*i+1*_ - *t*_*i*_, is applied around the chosen head axis, ***u*** (*t*_*i*_), centered in ***H***_*c*_(*t*_*i*_), translating and rotating the flying base of the motion platform.

Accordingly, the platform centroid position that ensures the correct head-centric vestibular stimulation is obtained by:

1) translating ***H***_*c*_(*t*_*i*_) to the origin of the motion platform system, so that the rotation axis ***u*** (*t*_*i*_) = ( $u_{x}^{S}\left(t_{i}\right),u_{y}^{S}\left(t_{i}\right),u_{z}^{S}\left(t_{i}\right)$ ) passes through the origin using

$$D\left(t_{i}\right)=\left[\begin{array}{l} \begin{array}{llll} 1 & 0 & 0\;-H_{cx}^{S}\left(t_{i}\right)\\ 0 & 1 & 0\;-H_{cy}^{S}\left(t_{i}\right)\\ 0 & 0 & 1\;-H_{cz}^{S}\left(t_{i}\right)\\ 0 & 0 & 0 & 0 \end{array} \end{array}\right]$$ (29)

2) rotating the flying base of the motion platform by the angle d*θ*(*t_i_*) around the axis *u*(*t_i_*) with

$$R\left(t_{i}\right)=\left[\begin{array}{c} \begin{array}{cccc} R_{11}\left(d\theta \left(t_{i}\right)\right) & R_{12}\left(d\theta \left(t_{i}\right)\right) & R_{13}\left(d\theta \left(t_{i}\right)\right) & 0\\ R_{21}\left(d\theta \left(t_{i}\right)\right) & R_{22}\left(d\theta \left(t_{i}\right)\right) & R_{23}\left(d\theta \left(t_{i}\right)\right) & 0\\ R_{31}\left(d\theta \left(t_{i}\right)\right) & R_{32}\left(d\theta \left(t_{i}\right)\right) & R_{33}\left(d\theta \left(t_{i}\right)\right) & 0\\ 0 & 0 & 0 & 1 \end{array} \end{array}\right]$$ (30)

$$\begin{array}{c} R_{11}\left(t_{i}\right)=\cos\left(d\theta \left(t_{i}\right)\right)+u_{x}^{2}*\left(1-\cos\left(d\theta \left(t_{i}\right)\right)\right)\\ R_{12}\left(t_{i}\right)=u_{x}\left(t_{i}\right)*u_{y}\left(t_{i}\right)*\left(1-\cos\left(d\theta \left(t_{i}\right)\right)\right)-u_{z}\left(t_{i}\right)*\sin\left(d\theta \left(t_{i}\right)\right)\\ \begin{array}{c} R_{13}\left(t_{i}\right)=u_{x}\left(t_{i}\right)*u_{z}\left(t_{i}\right)*\left(1-\cos\left(d\theta \left(t_{i}\right)\right)\right)+u_{y}\left(t_{i}\right)*\sin\left(d\theta \left(t_{i}\right)\right)\\ R_{21}\left(t_{i}\right)=u_{y}{\left(t_{i}\right)*u}_{x}\left(t_{i}\right)*\left(1-\cos\left(d\theta \left(t_{i}\right)\right)\right)+u_{z}\left(t_{i}\right)*\sin\left(d\theta \left(t_{i}\right)\right)\\ \begin{array}{c} R_{22}\left(t_{i}\right)=\cos\left(d\theta \left(t_{i}\right)\right)+u_{y}^{2}\left(t_{i}\right)*\left(1-\cos\left(d\theta \left(t_{i}\right)\right)\right)\\ \begin{array}{c} R_{23}\left(t_{i}\right)=u_{y}\left(t_{i}\right)*u_{z}\left(t_{i}\right)*\left(1-\cos\left(d\theta \left(t_{i}\right)\right)\right)-u_{x}\left(t_{i}\right)*\sin\left(d\theta \left(t_{i}\right)\right)\\ R_{31}\left(t_{i}\right)=u_{z}\left(t_{i}\right)*u_{x}\left(t_{i}\right)*\left(1-\cos\left(d\theta \left(t_{i}\right)\right)\right)-u_{y}\left(t_{i}\right)*\sin\left(d\theta \left(t_{i}\right)\right)\\ \begin{array}{c} R_{32}\left(t_{i}\right)=u_{z}{\left(t_{i}\right)*u}_{y}\left(t_{i}\right)*\left(1-\cos\left(d\theta \left(t_{i}\right)\right)\right)+u_{x}\left(t_{i}\right)*\sin\left(d\theta \left(t_{i}\right)\right)\\ R_{33}\left(t_{i}\right)=\cos\left(d\theta \left(t_{i}\right)\right)+u_{z}^{2}\left(t_{i}\right)*\left(1-\cos\left(d\theta \left(t_{i}\right)\right)\right) \end{array} \end{array} \end{array} \end{array} \end{array}$$

3) bringing ***H***_*c*_(*t*_*i*_) back to its initial position, that is, performing an inverse translation of that described at point 1) above (Eq. 29):

$$D^{-1}\left(t_{i}\right)=\left[\begin{array}{l} \begin{array}{llll} 1 & 0 & 0 & H_{cx}^{S}\left(t_{i}\right)\\ 0 & 1 & 0 & H_{cy}^{S}\left(t_{i}\right)\\ 0 & 0 & 1 & H_{cz}^{S}\left(t_{i}\right)\\ 0 & 0 & 0 & 1 \end{array} \end{array}\right]$$ (31)

that is:

$$p_{c}^{S}\left(t_{i+1}\right)=D^{-1}\left(t_{i}\right)*R\left(t_{i}\right)*D\left(t_{i}\right){*S}_{i}^{S}$$ (32)

with $S_{i}^{S}$ = [ $p_{{cx}^{s}}\left(t_{i}\right);p_{{cy}^{s}}\left(t_{i}\right)$ ; $p_{{cz}^{s}}\left(t_{i}\right)$ ; 1] corresponding to the position of the motion platform in *t = t*_*i*_

The platform orientation is given by the platform orientation in *t*_*i*_ (Rotational matrix ***O*** (*t*_*i*_)) following by the incremental rotation (***R*** (*t*_*i*_)):

$$R_{tot}\left(t_{i}\right)=R\left(t_{i}\right)*O\left(t_{i}\right)=\left[\begin{array}{c} \begin{array}{cccc} {R_{tot}}_{11}\left(t_{i}\right) & {R_{tot}}_{12}\left(t_{i}\right) & {R_{tot}}_{31}\left(t_{i}\right) & 0\\ {R_{tot}}_{21}\left(t_{i}\right) & {R_{tot}}_{22}\left(t_{i}\right) & {R_{tot}}_{32}\left(t_{i}\right) & 0\\ {R_{tot}}_{31}\left(t_{i}\right) & {R_{tot}}_{32}\left(t_{i}\right) & {R_{tot}}_{33}\left(t_{i}\right) & 0\\ 0 & 0 & 0 & 1 \end{array} \end{array}\right]$$ (33)

with

$$O\left(t_{i}\right)=O_{{Yaw}^{s}\left(t_{i}\right)}\;{*O}_{{Pitch}^{s}\left(t_{i}\right)}*O_{{Roll}^{s}\left(t_{i}\right)}=\left[\begin{array}{cccc} O_{11}\left(t_{i}\right) & O_{12}\left(t_{i}\right) & O_{13}\left(t_{i}\right) & 0\\ O_{21}\left(t_{i}\right) & O_{22}\left(t_{i}\right) & O_{23}\left(t_{i}\right) & 0\\ O_{23}\left(t_{i}\right) & O_{32}\left(t_{i}\right) & O_{33}\left(t_{i}\right) & 0\\ 0 & 0 & 0 & 1 \end{array}\right]$$ (34)

where:

$$\begin{array}{c} \begin{array}{c} O_{11}\left(t_{i}\right)=\cos\left({Yaw}^{s}\left(t_{i}\right)\right)*\cos\left({Pitch}^{s}\left(t_{i}\right)\right)\\ O_{12}\left(t_{i}\right)=\cos\left({Yaw}^{s}\left(t_{i}\right)\right)*\sin\left({Pitch}^{s}\left(t_{i}\right)\right)*\sin\left({Roll}^{s}\left(t_{i}\right)\right)-\sin\left({Yaw}^{s}\left(t_{i}\right)\right)*\cos\left({Roll}^{s}\left(t_{i}\right)\right)\\ O_{13}\left(t_{i}\right)=\cos\left({Yaw}^{s}\left(t_{i}\right)\right)*\sin\left({Pitch}^{s}\left(t_{i}\right)\right)*\cos\left({Roll}^{s}\left(t_{i}\right)\right)+\sin\left({Yaw}^{s}\left(t_{i}\right)\right)*\sin\left({Roll}^{s}\left(t_{i}\right)\right) \end{array}\\ \begin{array}{c} O_{21}\left(t_{i}\right)=\sin\left({Yaw}^{s}\left(t_{i}\right)\right)*\cos\left({Pitch}^{s}\left(t_{i}\right)\right)\\ O_{22}\left(t_{i}\right)=\sin\left({Yaw}^{s}\left(t_{i}\right)\right)*\sin\left({Pitch}^{s}\left(t_{i}\right)\right)*\sin\left({Roll}^{s}\left(t_{i}\right)\right)+\cos\left({Yaw}^{s}\left(t_{i}\right)\right)*\cos\left({Roll}^{s}\left(t_{i}\right)\right)\\ O_{23}\left(t_{i}\right)=\sin\left({Yaw}^{s}\left(t_{i}\right)\right)*\sin\left({Pitch}^{s}\left(t_{i}\right)\right)*\cos\left({Roll}^{s}\left(t_{i}\right)\right)-\cos\left({Yaw}^{s}\left(t_{i}\right)\right)*\sin\left({Roll}^{s}\left(t_{i}\right)\right) \end{array}\\ \begin{array}{c} O_{31}\left(t_{i}\right)=-\sin\left({Pitch}^{s}\left(t_{i}\right)\right)\\ O_{32}\left(t_{i}\right)=\cos\left({Pitch}^{s}\left(t_{i}\right)\right)*\sin\left({Roll}^{s}\left(t_{i}\right)\right)\\ O_{33}\left(t_{i}\right)=\cos\left({Pitch}^{s}\left(t_{i}\right)\right)*\cos\left({Roll}^{s}\left(t_{i}\right)\right) \end{array} \end{array}$$

so:

$${Roll}^{s}\left(t_{i+1}\right)=\mathrm{atan}2\left({R_{tot}}_{32}\left(t_{i}\right),{R_{tot}}_{33}\left(t_{i}\right)\right)\;{Pitch}^{s}\left(t_{i+1}\right)=-\mathrm{asin}\left({R_{tot}}_{31}\left(t_{i}\right)\right)\;{Yaw}^{s}\left(t_{i+1}\right)=\mathrm{atan}2\left({R_{tot}}_{21}\left(t_{i}\right),{R_{tot}}_{11}\left(t_{i}\right)\right)$$ (35)

Thus, the overall transformation of the POSE of the platform that guarantees a rotation of *θ(t)* about the head axis ***u*** that passes through the ***H***_*c*_ is given by Eqs. 32–35.

## 5 Feasibility check of the stimulation profile

Before executing the vestibular stimulation motion profile (Eq. 28 for translations or Eqs 32–35 for rotations), we record the head position and orientation and we simulate the entire selected motion profile with that head configuration. The simulation is necessary to verify the feasibility of the motion profile, and prevent the platform from stalling midway. In the feasibility check, we consider that *u*(*t*) = *u*(*t* = 0). The limits of lengthening and shortening of the platform actuators constrain the range of motion. The feasibility check involves the measurement of the elongation of the actuators during the execution of the motion profile. Starting from a specified motion profile of the platform (in terms of position and orientation), we solve the inverse kinematic problem to estimate the corresponding time-history of length changes of the six actuators. The presented algorithm is based on the geometrical approach by Hulme and Pancotti (2014). If the motion profile is not compatible with the physical limits of the actuators, an iterative algorithm is used to find a “better-starting position” of the platform. If even the “better-starting position” does not guarantee a compatible motion, the specific motion profile is not further considered for execution.

**FIGURE 4:**
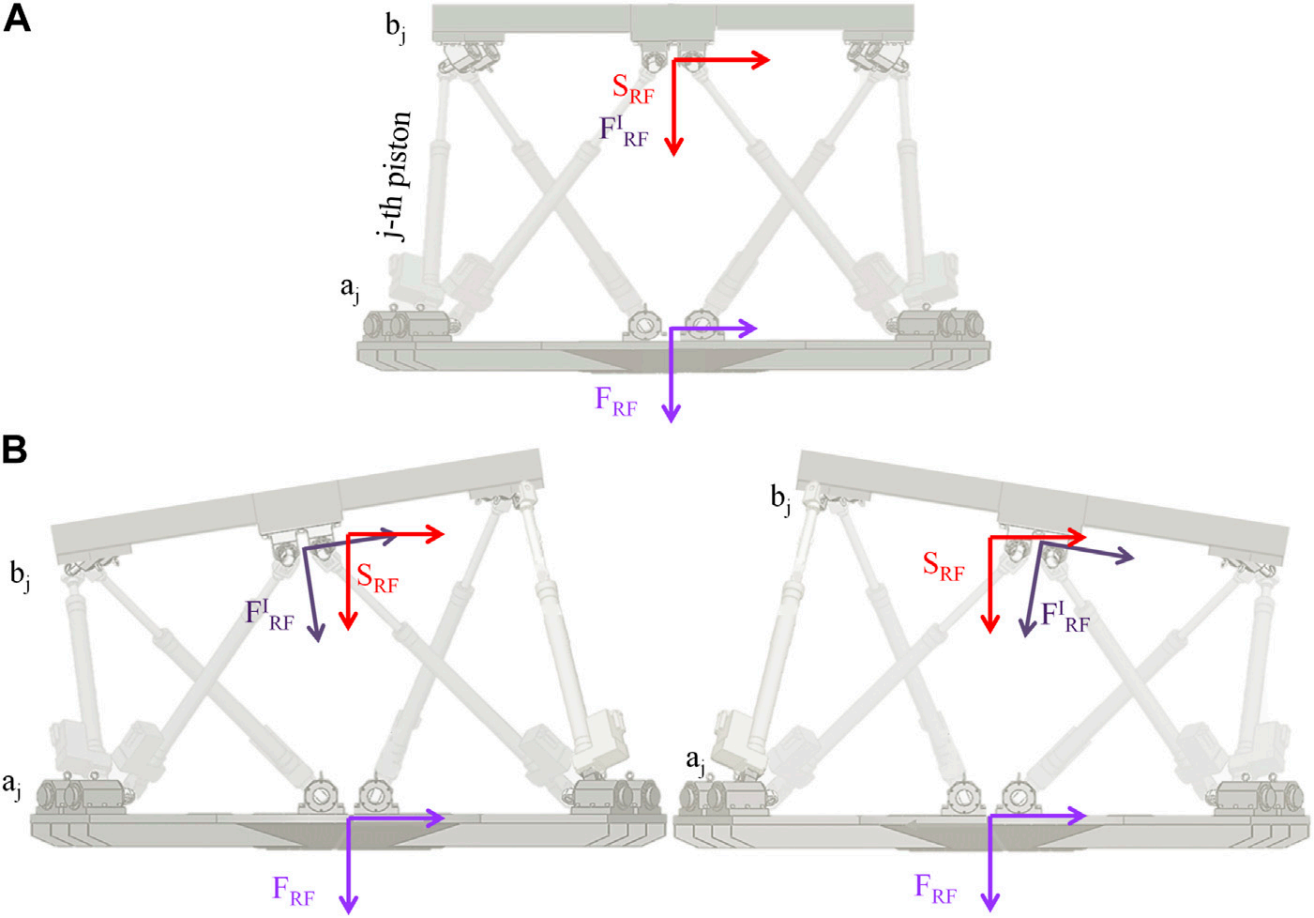
Moving platform reference frames. In **(A)** the platform is in REST position (all fully retracted actuators). The fixed reference frame (F_RF_) affixed to the base of the motion platform, the moving reference frame (F^I^_RF_) affixed to the flying base of the motion platform and the Stewart platform Fixed reference frame (S_RF_) are shown in light violet, dark violet and red respectively. In REST position F^I^_RF_ and S_RF_ are coincident. **(B)**, the same format of **(A)**, but the platform is in two different POSEs.

Consider a fixed reference frame (F_RF_) aligned with the base of the motion platform, and a moving reference frame (F^I^_RF_) aligned with the flying base of the motion platform centered in ***p***_*c*_ (see Figure 4). The position of the origin of F^I^_RF_ with respect to that of F_RF_ is defined by the vector:

$${r\left(t\right)}_{\text{FIXED}}=r_{\text{FIXED}\_\text{REST}}+\left(p_{cx}^{S}\left(t\right),p_{cy}^{S}\left(t\right),p_{cz}^{S}\left(t\right)\right)$$ (36)

$r_{\text{FIXED}\_\text{REST}}$ is the vector representing the distance between the two origins when the platform is in its REST position (all actuators fully retracted), corresponding to no antero-posterior, lateral, or vertical displacement, and no rotation. This vector is provided in the user manual of the motion platform. In fully retracted actuators, F^I^_RF_ is coincident with S_RF_ (Figure 4A).

The contact point of the *j*th actuator with the flying base of the motion platform (***b***_***j***_) can be expressed in F_RF_ as:

$$b_{j}^{F}\left(t\right)={r\left(t\right)}_{\text{FIXED}}+O\left(t\right)*b_{j}^{F^{I}}\left(t\right)$$ (37)

$b_{j}^{F^{I}}$ is the same contact point in F^I^_RF_ coordinates, and $O\left(t\right)$ the rotation of the platform (F^I^_RF_) with respect to the F_RF_, function of the user-specified *Roll* ^*S*^, *Pitch* ^*S*^, and *Yaw* ^*S*^ of the flying base (Eq. 34).

The *j*th actuator length is computed as the length of the vector connecting the *j*th piston contact point with the base of the motion platform ( $a_{j}^{F}$ ) to that with the upper base ( $b_{j}^{F}$ )

$${\text{Act}}_{j\_\text{length}}=\left\|b_{j}^{F}\left(t\right)-a_{j}^{F}\right\|=\left\|{r\left(t\right)}_{FIXED}+Q\left(t\right){*b}_{j}^{F^{I}}\left(t\right)-a_{j}^{F}\right\|$$ (38)

with *j* = 1:6, number of actuators.

This calculation is done for the entire motion profile, in order to have the length of every actuator, from the base contact points to those of the flying frame, over the entire movement duration. The elongation is then evaluated by subtracting the fully retracted actuator length ( ${\text{Act}}_{j\_\text{length}\_\text{REST}}$ ) from the computed length in the motion profile simulation. ${\text{Act}}_{j\_\text{length}\_\text{REST}}$ is obtained with the above mentioned equations, knowing that the REST condition refers to zero rotation, zero translation of the platform. The rotation matrix ***Q*** corresponds to the identity matrix, and the vector $r_{\text{FIXED}}$ corresponds to $r_{\text{FIXED}\_\text{REST}}$ .

Finally, the actuator elongation is:

$${\text{Act}}_{{j\_}_{\text{elong}}}\left(t\right){=\text{Act}}_{j\_\text{length}}\left(t\right){-\text{Act}}_{j\_\text{length}\_\text{REST}}$$ (39)

The movement can be performed if the computed elongation for each motion instruction of the profile falls within the allowable range [L_*j*_min_-L_*j*_max_] of the actuators. Alternatively, the following approach is used to find a “better-starting point” of the motion platform centroid that is suitable for the specified motion profile:

1. Relocate the initial position of $p_{c0}^{S}$ to the center of motion platform workspace
2. Evaluate the motion profile that starts from the new initial position
3. Calculate the elongation of each actuator (Eq. 39)
4. Check the movement feasibility
5. If the new motion is still incompatible with the actuators physical constraints, repeat steps 2–4 after shifting the initial position ( $p_{c0}^{S}$ ) inside the system workspace
6. If the motion remains incompatible after exploring the whole motion system workspace (changing the position of $p_{c0}^{S}$ ), the specific motion profile is rejected and not executed. Alternatively, the position of the participant on the motion platform system can be changed.

## 6 Exemplary applications of head-centric stimulations under head-free conditions

To assess the vestibular function during passive head and body movements, one can restrain the participant’s head in a fixed position (for instance, with a helmet or tight forehead strap) and then output command motion signals to the platform relative to that fixed reference frame. Here, we consider the alternative option of asking participants to freely choose a comfortable position of their unrestrained head, and then not to move the head voluntarily during the stimulation. Importantly, participants are not asked to stiff their neck to keep the head stationary, but only to avoid moving the head on purpose. Since we monitor head position and orientation during the stimulation, we can verify either off-line or on-line by how much the head moved. There are two different approaches to achieve head-centric stimuli under head-free conditions, depending on whether one wants to account for head movement during the trial off-line or on-line.

The first solution (off-line) involves a “*Fixed head-reference-fram*e.” In this case, one can generate the desired head-centric motion profile (POSE (t)) in each trial by using the head-centered reference frame evaluated at the start of the trial. To this end, one applies Eq. 28 for translations (with ***u*** (*t*_*i*_) = ***u*** (*t* = 0)) and Eqs 32–35 for rotations (with ***u*** (*t*_*i*_) = **u**(*t* = 0) and ***H***_***c***_(*t*_*i*_) = ***H***_***c***_(*t* = 0)). For a given POSE(*t*), the Central Control Unit sends the instantaneous desired POSE(*t*_*i*_) to the platform controller while also recording platform feedback. Simultaneously, the Central Control Unit acquires 3D position and orientation of the head and platform from the motion capture system (see Figure 2). During platform motion, a running average of 3D head position and orientation over 50-ms consecutive intervals is computed. If the head shifts (in x, y or z) or rotates (in roll, pitch, or yaw) relative to the chair (and platform) by more than a predefined tolerance window relative to the platform over any 50-ms interval, the trial can be discarded and repeated (La Scaleia et al., 2023). The tolerance window is specified *a priori* based on the task and the participant’s characteristics (for instance, the tolerance may be greater for patients with significant cognitive or sensorimotor impairments).

The second solution (on-line) involves a “*Moving head-reference-frame*.” In this case, the desired head-centric motion profile (POSE(*t*)) is created using the position (***H***_***c***_(*t*_*i*_)) and orientation of the head (***u*** (*t*_*i*_)) measured on-line during the trial (using Eq. 28 for translations and Eqs 32, 35 for rotations). This last solution is more general than the first solution, and can be used to test vestibular function both when the participant keeps the head stationary relative to the chair and when the participant moves the head voluntarily during externally applied motions.

## 7 Algorithms validation

We implemented and validated the algorithms using the two approaches mentioned above (*Fixed* and *Moving head-reference-frame*) with a MOOG MB-E-6DOF/12/1000 Kg (East Aurora, New York, United States) as Stewart Platform. We used Vicon 3D Motion Capture system (Vicon Motion Systems Ltd., United Kingdom), equipped with 10 IR Vero 2.2 cameras, to track head and platform motion in real time. In our set-up, the MOOG is controlled in *Degrees Of Freedom* mode by a server (Central Control Unit) that implements the UDP communication with the motion platform at 1 kHz (freq_command) according to the interface manual (CDS7330- MOOG Interface Definition Manual For Motion Bases). Moreover, the server opens a UDP socket allowing the connection with the motion capture system. All position profiles were programmed in LabVIEW 2023 (National Instruments, Austin, Texas, United States) with custom-written software, and input to the MOOG controller at 1 kHz. 3D data provided by the motion capture system were sampled at 200 Hz. Also the platform feedback was acquired at 200 Hz. Only for the spatial calibration (see Sections 2.2.1; 2.2.2), the platform feedback was acquired at 1 kHz. In order to find the corresponding paired points (from the Stewart Platform and the motion capture system, respectively) to include in the *Least-Squares Fitting of Two 3D Point Sets* algorithm (Arun et al., 1987), we interpolated the data collected with the motion capture system at 1 kHz using the Matlab function *interp1*, with the ‘spline’ method.

In our tests, we used single cycles of sinusoidal acceleration, consistent with several previous studies of vestibular motion perception (e.g., Benson et al., 1986; Valko et al., 2012; Bermúdez Rey et al., 2016; Bremova et al., 2016; Kobel et al., 2021a; La Scaleia et al., 2023). We restricted our tests to translational motions, but our approach can be easily applied to rotational motions or a combination of translations and rotations.

The equations defining a translation along a cardinal axis ***u*** (interaural, naso-occipital or cranio-caudal) are as follows:

$$a\left(t\right)=A*\sin\left(2\pi ft\right)$$ (40)

$$v\left(t\right)=AT_{\text{norm}}\left[1-\cos\left(2\pi ft\right)\right]$$ (41)

$$s\left(t\right)=\left({\text{AT}}_{\text{norm}}\right)\left[t-T_{\text{norm}}\sin\left(2\pi ft\right)\right]$$ (42)

Acceleration (*a(t)*), speed (*v(t)*), and position (*s(t)*) are all proportional to each other. We set the sinusoidal acceleration frequency at f = 0.2 Hz, the duration of the motion cycle at T = 5 s and T_norm_ = $T/\left(2\pi \right)$ . We set the peak of velocity *v*_*p*_ = 2AT_norm_ = 0.04 m/s, and we obtained the amplitude of acceleration A = 0.025 m/s^2^ and a total translation *s*(t = T) = 0.10 m.

For the condition with “*Fixed head-reference-frame*,” we monitored a participant seated in the padded racing chair. During each trial, the person kept the head in a resting position, which was otherwise unrestrained from the outside. The motion platform was translated naso-occipitally (100 trials) by using the ***NO*** head axis that was assessed at the start of each trial. We tracked the 3D position and orientation of the head and platform by means of Vicon. To this end, we attached 4 markers at the chair and 6 markers at the head, i.e., the 5 markers of Figure 3, plus one in an arbitrary position to produce asymmetry (M_6_). For our tests, we chose the left pterion for this additional marker. The POSE of the motion platform (motion command and feedback), as well as the position and orientation of the head and chair, were all recorded synchronously at 200 Hz.

For the condition with “*Moving head-reference-frame*,” we performed two different experiments. First, we evaluated the delays inherent in the on-line algorithm implementation. In order to have a highly controlled condition with the absence of head movements, we firmly attached a mannequin head to the headrest of the platform chair, which was then translated along inter-aural, naso-occipital or cranio-caudal directions. The mannequin head was fixed in a position roughly corresponding to that of the head of participants in typical tests of vestibular motion discrimination (e.g., La Scaleia et al., 2023). We then controlled the motion platform using the head orientation monitored in real time. Specifically, we tracked the 3D position and orientation of the mannequin head (with the same markers’ position as in the previous condition) and the platform at 200 Hz using Vicon. Using Eqs. 26, 28 and Eq. 41, the motion platform was controlled on-line after choosing the head axis for stimulation ( $u_{NO}$ or $u_{IA}$ or $u_{CC}$ ). We performed 100 trials for each head axis (***NO***, ***IA***, ***CC***). Thus, in contrast with the “*Fixed head-reference-frame*” condition, here the head axis was time-varying, being computed on-line during each trial.

We then carried out the same procedure used with the mannequin head on a head-free participant seated in the chair. In this experiment, the task for the participant was to move the head smoothly in order to explore the surrounding. In each trial, while the participant shifted the head, we applied a naso-occipital translation of the motion platform (100 trials) by using the ***NO*** head axis that was assessed in real-time during the trial (Eqs. 26, 28 and Eq. 41).

### 7.1 Data analysis

The 3D coordinates (x, y, z) of the motion platform recorded by the motion platform system (command and feedback data), as well as the 3D coordinates of the chair and the head (mannequin or real) recorded by Vicon were numerically low-pass filtered (bidirectional, first-order Butterworth filter, 15 Hz cut-off). For each trial, we computed the head orientation (***u*** *(t)*) and the first time derivative of the platform command ( $v_{S_{command}}$ (*t*)), the platform feedback ( $v_{S_{FB}}\left(t\right)$ ), the chair motion ( $v_{Chair}\left(t\right)$ ), and the head motion ( $v_{Head}\left(t\right)$ ). We then calculated the projection of these motion profiles on the selected head axis (***u***):

$${v_{S_{command}}^{u}\left(t\right)=v}_{S_{command}}\left(t\right)∙u\left(t\right)$$ (43)

$${v_{S_{FB}}^{u}\left(t\right)=v}_{S_{FB}}\left(t\right)∙u\left(t\right)$$ (44)

$${v_{Chair}^{u}\left(t\right)=v}_{Chair}\left(t\right)∙u\left(t\right)$$ (45)

$${v_{Head}^{u}\left(t\right)=v}_{Head}\left(t\right)∙u\left(t\right)$$ (46)

To assess how well the actual motion profiles matched the desired motion profile, for each trial we computed the cross-correlation between the desired motion profile of the platform (Eq. 41) and the command motion profiles (Eq. 43), and between the latter and the actual motion profiles executed as recorded by the MOOG feedback (Eq. 44) or by the Vicon. For the latter, we considered both the chair motion (Eq. 45) and the head motion (Eq. 46).

For the condition “*Static head-reference-frame*,” we computed the time lag as the time instant when the absolute value of the cross-correlation was maximum (with a 5 ms resolution, due to 200 Hz sampling frequency). A positive time lag indicates a delay of the actual motion with respect to the reference motion. We also defined the coefficient of cross-correlation (CoefCC) as the value of the cross-correlation at the time lag. The normalized coefficient of cross-correlation (NCC) was then calculated as CoefCC divided by the coefficient of autocorrelation of the desired motion profile (Eq. 41) at zero lag. The value of NCC indicates the degree of similarity between the two compared motion profiles: the closer the NCC is to one, the greater the similarity.

For the condition “*Dynamic head-reference-frame*,” we first computed the NCC and the time lag inherent the on-line algorithm implementation using the fixed mannequin head. In this manner we evaluated the time lag that represents the cumulative delay of the algorithm implementation and the overall system (i.e., Motion Platform, Motion Capture). Then, we assessed the NCC with the moving head of the participant using the time lag evaluated with the fixed mannequin head in order to evaluate the quality of the provided stimulus, taking into account the algorithm’s inherent delay, in the presence of the participant’s voluntary head movement.

### 7.2 Statistical analysis

Statistical analyses were performed in Matlab 2021b. We used the Shapiro–Wilk test to verify the normality of distribution of data, and we found that the data were not normally distributed (see Results below). Accordingly, we report median values and 95% confidence intervals (CI). We used non-parametric statistics (Friedman test) to evaluate if the command motion profiles computed with the proposed algorithm (POSE, Eq. 28) depend on the selected head axis (***NO***, ***IA***, ***CC***).

## 8 Experimental results

### 8.1 Static head-reference-frame

In each trial of this experiment (100 trials), the participant kept the unrestrained head in a resting, comfortable position. The motion platform was translated naso-occipitally by using the ***NO*** head axis assessed at the start of each trial. The median head orientation, in S_RF,_ at trial start was 180.358°, 7.847°, −3.054° {95% CI (180.271°-180.446), (7.600°–8.095°), [(-3.388°)-(-2.720°)], *n* = 100} in roll, pitch and yaw, respectively. During the platform motion, the maximum head rotation relative to the head orientation evaluated at the start of the trial was 1.045°, 2.186° and 2.209° in roll, pitch and yaw, respectively. The median value of NCC between the desired motion profile of the platform (Eq. 42) and the command motion profiles (Eq. 43) was 0.997 [95% CI 0.997; 0.997].

Table 1 reports the median (and 95% CI) of the normalized correlation coefficients (NCC) and time lags between the command motion profiles (Eq. 43), and the actual motion profiles executed as recorded by the MOOG feedback (Eq. 44) or by the Vicon. For the latter, we considered both the chair motion (Eq. 45) and the head motion (Eq. 46). The high values of NCC show the excellent agreement between the theoretical profile of the motion stimuli and the corresponding profile of actual motion as measured in real-time. The measured value of time lag (about 95 ms) between the command and feedback is intrinsic to the specific platform hardware we used (see specifications at Section 7) and is independent of the algorithm (Figure 2). This lag roughly agrees with the value of 117 ms reported by Huryn et al. (2010), who communicated with the platform at 60 Hz. The motion capture processing in our system introduces some additional 5 ms delay in the estimate of time lags measured at the chair and head.

**TABLE 1:** Static head-reference-frame: median [and (95% CI)] cross-correlation coefficient between the speed motion command profile and the actual speed motion profile (platform feedback, chair and head).

Motion Profile	Platform feedback Motion profile		Chair motion profile		Head motion profile	
	Normalized cross-correlation	Time-lag [ms]	Normalized cross-correlation	Time-lag [ms]	Normalized cross-correlation	Time-lag [ms]
***NO***	0.994 [0.994–0.994]	95 [95–95]	1.006 [1.006–1.007 ]	100 [100–100]	1.014 [1.014–1.015]	100 [99–101]

### 8.2 Dynamic head-reference-frame

#### 8.2.1 Fixed mannequin head

The head orientation in S_RF_ at the start of the trial and the maximum head rotation relative to the initial head orientation during the motion platform are shown in Table 2.

**TABLE 2:** Dynamic head-reference-frame with fixed mannequin head: median [and (95% CI)] head orientation in S_RF_ at the start of the trial and maximum head rotation, during the motion platform, relative to the head orientation at the start of the trial.

Motion profile	Head orientation at the start of the trial			Maximum head rotation		
	Roll [°]	Pitch [°]	Yaw [°]	Roll [°]	Pitch [°]	Yaw [°]
***NO***	169.959 [169.958–169.961]	1.202 [1.200–1.204]	−28.639 [(-28.642)-(-28.637)]	0.086	0.179	0.080
***IA***	170.193 [170.180–170.206]	1.322 [1.314–1.329]	−28.683 [(-28.685)-(-28.681)]	0.145	0.068	0.107
***CC***	169.991 [169.985–169.996]	1.194 [1.190–1.197]	−28.617 [(-28.620)-(-28.614)]	0.376	0.183	0.157

**FIGURE 5:**
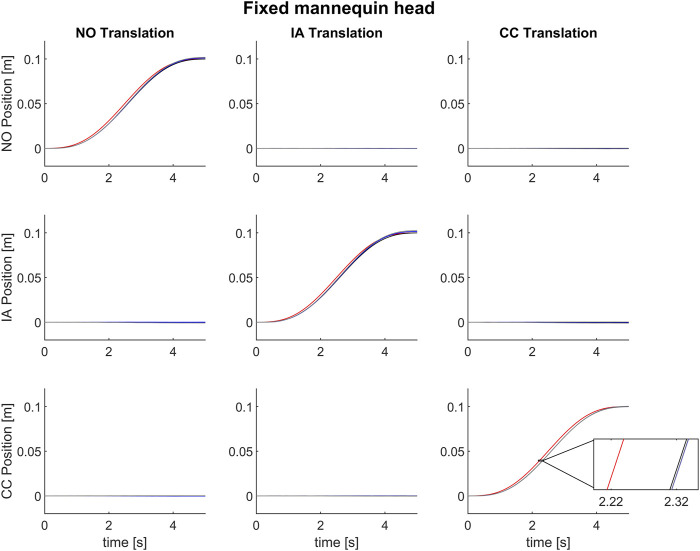
Mean motion profiles along the naso-occipital (top row), inter-aural (middle row) and cranio-caudal axis (bottom row) using the control “Dynamic head-reference-frame.” The motion stimulus consisted of a single cycle of 0.2 Hz sinusoidal acceleration in the naso-occipital (first column), inter-aural (second column) or cranio-caudal direction (third column) in the Stationary head condition. The red line represents the motion command sent to the motion platform. The black, blue, and gray lines show the executed motion in terms of platform feedback, chair and head data (as recorded by the motion tracking system). In the magnified inset notice a lag of about 95 ms between the command signal and the recorded motions by platform feedback, and of about 5 ms between executed motion in terms of platform feedback and the data recorded by motion capture system.

Figure 5 displays the mean motion profiles that were obtained over 100 trials along the head axes during stimulation in the ***NO***, ***IA***, or ***CC*** directions. Motion command is in red, while executed motions as revealed by the platform feedback, chair and head are in black, blue, and gray, respectively. One can notice the excellent agreement between the command and the actual motion, except for a time lag.

We found a high correlation between the desired motion profile of the platform (Eq. 41) and the command motion profiles (Eq. 43) evaluated using the on-line orientation of the mannequin head. NCC values were not normally distributed for any of the 3 motion directions (***NO***, ***IA***, ***CC***, all *p*-values <0.001, Shapiro–Wilk test). The NCC did not depend significantly on the translation direction (***NO***, ***IA*** or ***CC***) (Friedman test χ^2^(2) = 1.14, *p* = 0.5655). The median value of NCC between the desired motion profiles and the command motion profiles across all trials and tested translation directions was 0.997 [95%CI 0.997; 0.997].

Table 3 reports the median (and 95% CI) of the normalized correlation coefficients (NCC) and time lags in the same format as in Table 1. Both sets of values are comparable to those obtained in the condition *Static head-reference-frame*, indicating that the procedure of estimating on-line the position and orientation of the mannequin head is robust and reliable.

**TABLE 3:** Dynamic head-reference-frame with fixed mannequin head: median [and (95% CI)] cross-correlation coefficient between the speed motion command profile and the actual speed motion profile (platform feedback, chair and head).

Motion Profile	Platform feedback motion profile		Chair motion profile		Head motion profile	
	Normalized cross-correlation	Time-lag [ms]	Normalized cross-correlation	Time-lag [ms]	Normalized cross-correlation	Time-lag [ms]
***NO***	0.994 [0.994–0.994]	95 [95–95]	1.006 [1.005–1.006]	100 [100–100]	1.013 [1.013–1.013]	100 [99–101]
***IA***	0.994 [0.994–0.994]	95 [95–95]	1.009 [1.009–1.009]	100 [100–100]	1.018 [1.018–1.019]	100 [100–100]
***CC***	0.993 [0.993–0.993]	95 [95–95]	0.996 [0.996–0.996]	100 [100–100]	0.995 [0.995–0.995]	100 [100–100]

#### 8.2.2 Moving head of the participant

**FIGURE 6:**
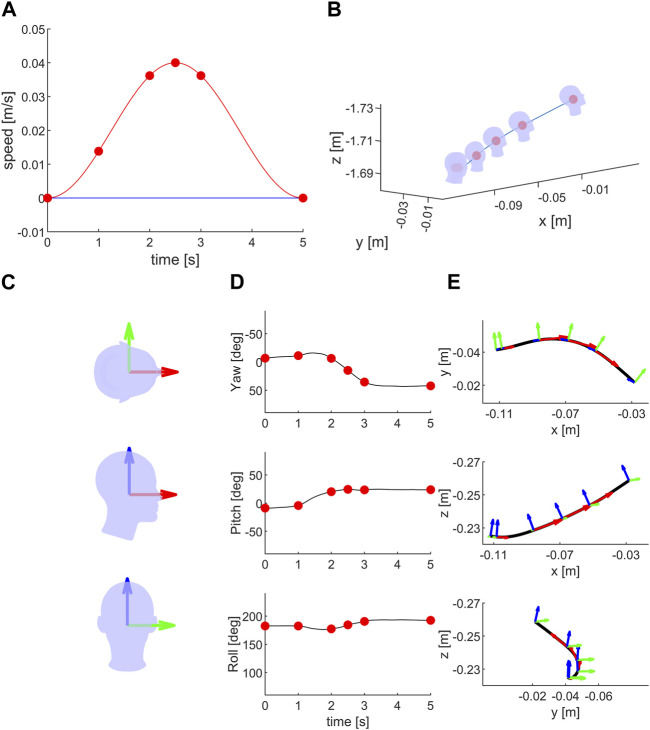
Moving head during NO translation: **(A)** Ideal stimulus: speed motion profile along the naso-occipital, inter-aural and cranio-caudal axes in red, green and blue lines, respectively. Green and blue lines are overlapped. Red dots represent the speed values in NO direction during the motion (after 1, 2, 2.5, 3 and 5 s from the start). **(B)** The head passive movement during the stimulation (blue line) in a single trial in S_RF_. The head orientation during the passive motion are shown at the start of the stimulation and at the same time of red dots of **(A)** The head schematic is not drawn to scale (∼1:15) relative to the passive movement (blue line). **(C)** Head and naso-occipital (red), inter-aural (green) and cranio-caudal (blue) axes in top, lateral and front views. **(D)** Head orientation, yaw, pitch and roll, during the same trial as in **(B)**. **(E)** Black line represents the recorded platform movement, while red, green and blue arrows represent the head orientation during the platform motion as for **(B)**. The length of the red arrow is proportional to the stimulus speed **(A)**. The platform movement was updated online using the head orientation recording. The platform movement is tangential to the NO axis (for the head axes definition see Figure 3).

Figure 6 shows an example of the condition in which the platform was translated along the ***NO*** axis while the participant moved the head voluntarily in arbitrary directions during the trial. The effect of head voluntary orientation during the passive translation in 3D Cartesian space can be appreciated in panel B. Panel D depicts the time changes of head orientation in 3D angular space associated with the voluntary rotation. Panel E depicts the platform path as well as the orientation of the head frame (H_RF_^S^) in top, lateral, and front views.

Table 4 reports the median (and 95% CI) of the normalized correlation coefficients (NCC). Even in this case, one can notice the excellent agreement between the commanded motion stimuli and the actual motion as measured on-line.

**TABLE 4:** Dynamic head-reference-frame with moving head of the participant: median [and (95% CI)] cross-correlation coefficient between the speed motion command profile and the actual speed motion profile (platform feedback, chair and head).

Motion Profile	Platform feedback motion profile	Chair motion profile	Head motion profile
	Normalized cross-correlation at time-lag 95 ms	Normalized cross-correlation at time-lag 100 ms	Normalized cross-correlation at time-lag 100 ms
***NO***	0.992 [0.992–0.993]	1.005 [1.004–1.005]	1.074 [1.056–1.093]

## 9 Conclusion and perspectives

We have detailed and validated the algorithms to generate arbitrary head-centered motion stimuli in 3D under head-free conditions. Motion stimuli were produced by a MOOG platform. We tested these algorithms using a *Static* or a *Dynamic head-reference-frame*. The head was stationary relative to the platform or it moved throughout the platform translation. In all tested cases, we found excellent agreement between the theoretical profile of the motion stimuli and the corresponding profile of actual motion as measured in real-time.

It is worth being mentioned that any deviation between the theoretical profile and the actual profile of the stimuli should be assessed vis-à-vis the specific behavioral outcome being tested. For instance, if the test involves the estimate of vestibular thresholds, no corrections in the stimuli implementation are needed if the errors are smaller than the variability in the thresholds.

Our approach lends itself to vestibular tests that require head-free conditions, either because head-restraints are not recommended or because the test involves voluntary or reflex head movements during the platform motion stimuli. Head-free testing is especially relevant in the light of the growing interest in the exploration of the vestibular function under ecological conditions (Carriot et al., 2014; Diaz-Artiles and Karmali, 2021; La Scaleia et al., 2023; Lacquaniti et al., 2023). Indeed, it is now known that the vestibular system performs best when faced with naturalistic inputs, such as those encountered during head-free conditions (Cullen, 2019; Carriot et al., 2022; Sinnott et al., 2023).

Body-centric computations, such as the head-centric algorithms we presented, are becoming crucial to explore human physiology from the subjective perspective of each individual, as well as for personalized health monitoring. We can also envisage applications of our algorithms in novel virtual realities for flight or driving simulators that aim at minimizing the probability of motion sickness (Groen and Bos, 2008).

From a diagnostic point of view, it can be very useful to administer vestibular stimuli with specific orientations with respect to semicircular canals rather than stimulating the vestibular system with rotational stimuli around cardinal axes of the head. The availability of individual anatomical high-resolution magnetic resonance images of the head makes it possible to determine the specific orientation of the semicircular canals with respect to the Frankfurt plane. The proposed methodology allows to administer vestibular stimuli in any direction and around any predefined axis with respect to the head (within the range of movement of the platform), then it allows also to administer vestibular stimuli around specific semicircular canals’ axes of rotations. The evaluation of vestibular perception of vestibular stimuli centered on the individual vestibular system can certainly facilitate the diagnosis of functional abnormalities of specific semicircular canals.

Future developments of our approach will involve compensation strategies to reduce the current delay between the command and the actual motion in our system. Although in the *Dynamic head-reference-frame* condition the motion profile was evaluated instantaneously using the on-line head orientation, it was executed after ∼100 ms. Most of this delay (about 95 ms) was intrinsic to the specific platform hardware that we used (the remaining 5 ms depended on our motion capture system). In general, the presence of substantial delays in the system poses limits to the kind of head movements that can be taken into account in the *Dynamic head-reference-frame* condition. Indeed, the participant who tested this condition moved the head smoothly and relatively slowly, as it typically occurs in smooth pursuit tracking of visual targets. Fast head movements, such as those involved in head saccades, would not be corrected in time to adjust the estimates of head axes reliably. To address this limitation, we can envisage either using faster motors driving a Stewart platform or predictive algorithms to extrapolate the current measurements of head position and orientation into the future (e.g., autoregressive or Kalman filter models). For instance, a lead-compensation strategy based on a heuristically tuned linear prediction of the desired position of the MOOG platform reduced the delay between the desired and actual position to 33 ms in a study of human balance (Luu et al., 2011), involving data communication with the platform at 60 Hz.

## Figures and Tables

**Table:** 

^	Maximum likelihood estimate of the variable
CoefCC	Coefficient of Cross-Correlation
***CC***	Head-fixed Cranio-Caudal axis
FP	Frankfurt Plane
freq_command	Update rate of motion command and feedback in Stewart platform communication
F_RF_	Fixed reference frame affixed to the base of the motion platform
$F_{\text{RF}}^{I}$	Moving reference frame affixed to the flying base of the motion platform
H_c_	Center of the head
H_RF_	Head Reference Frame: head-fixed axes
***IA***	Head-fixed Inter-Aural axis
MC	Motion Capture system
MC_RF_	Motion Capture system reference frame
***n***	Unit normal vector orthogonal to the Frankfurt plane
***N***	Noise Vector
NCC	Normalized Coefficient of Cross-correlation
***NO***	Head-fixed Naso-Occipital axis
***O***	Rotation matrix of the Stewart platform
***p***_***c***_	Centroid of the Stewart platform’ flying base relative to S_RF_
$p_{i}^{MC}$	Point “p_i_” in MC_RF_
$p_{i}^{S}$	Point “p_i_” in S_RF_
POSE	Position and orientation of the Stewart platform’ flying base relative to the reference frame S_RF_.
***R***	Matrix that rotates the flying base of the motion platform by the angle *θ* around the axis ***u***
$R_{MC}^{S}$	Rotation matrix to map a point represented in the reference frame MC_RF_ to a point represented in the reference frame S_RF_
S_RF_	Stewart platform reference frame located in the flying base (motion) centroid when the flying base is in its home settled position. Motion centroid is the centroid of the joints below the flying base of the Stewart platform. When the flying base is at home all actuators are fully retracted into the mechanical stops. X-Y plane is the plane that fits, in a least squares sense, the joints below the flying frame. *X*, *Y*, *Z*-axes’ orientation of S_RF_ is depicted in Figure 4
$T_{MC}^{S}$	Translation vector to map a point represented in the reference frame MC_RF_ to a point represented in the reference frame S_RF_
***u***	Unit vector of head-fixed axes
*x* ^*MC*^, *y* ^*MC*^, *z* ^*MC*^	x, y, z coordinates in MC_RF_
*x* ^*S*^, *y* ^*S*^, *z* ^*S*^	x, y, z coordinates in S_RF_
*Roll* ^*S*^, *Pitch* ^*S*^, *Yaw* ^*S*^	Rotation roll, pitch, yaw in S_RF_
Vectors and matrices are denoted by boldface letters	

## Data Availability

The raw data supporting the conclusion of this article will be made available by the authors, without undue reservation.
