# Supplementary material for: Head-centric computing for vestibular stimulation under head-free conditions
Source: Front Bioeng Biotechnol. 2023 Dec 7;11:1296901. doi: 10.3389/fbioe.2023.1296901 (PMC10734306; doi:10.3389/fbioe.2023.1296901)
Supplement: Supplementary file 1 [file Presentation1.PDF]

Appendix

S.1 Temporal Alignment of data obtained from the platform feedback and from the motion capture system

Given a possible temporal misalignment between the start movement command sent to the motion platform, the motion platform movement execution, the motion platform acquisition and the MC data acquisition, we use a subset of data (4 complete cycles) starting from the first peak of the sinewave for each recorded signal. In this way we can temporally align the signals (obtained from the platform feedback and from the motion capture system), and obtain the corresponding paired data from the two systems (see Figure S.1).

To identify the sample corresponding to the first peak of the sinewave signals the following approach is used:

1. Evaluate the travelled distance ($\boldsymbol{TD}$) by $\boldsymbol{p_c}$ (in S_{RF}) and by Marker(s) (in MC_{RF}) as follows:

$$\boldsymbol{TD}_{point}(i) = \sqrt{\left(x_{point}(i) - x_{point}(1)\right)^2 + \left(y_{point}(i) - y_{point}(1)\right)^2 + (z_{point}(i) - z_{point}(1))^2}$$

where i is the number of sample ($1 \leq i \leq$ total number of samples and *point* can be either $\boldsymbol{p_c}$ (in S_{RF}) or marker(s) (in MC_{RF})

2. Identify the first sample (${i_1}^{point}$) in which $\boldsymbol{TD}_{point} > 2.5 * SD(\boldsymbol{TD}_{point})$, with SD the standard deviation
3. Starting from $\boldsymbol{TD}_{point}({i_1}^{point})$ select the first sample (${i_2}^{point}$) in which $\boldsymbol{TD}_{point} < SD(\boldsymbol{TD}_{point})$,
4. Starting from $\boldsymbol{TD}_{point}({i_2}^{point})$ and going back to ${i_1}^{point}$ select the first sample (${i_3}^{point}$) in which $\boldsymbol{TD}_{point} > 2.5 * SD(\boldsymbol{TD}_{point})$
5. The sample corresponding to the first peak of the sinewave signal (i_{start}^{point}) is the average value between ${i_1}^{point}$ and ${i_3}^{point}$ rounded down.

$\boldsymbol{p_c}{}_i^S$ and $\boldsymbol{p}_{Marker}{}_i^{MC}$ (with $i_{start}^{point} \leq i \leq i_{start}^{point}$+4 cycles * number of samples in a single cycle) are the temporal aligned position of $\boldsymbol{p_c}$ (in S_{RF}) and of Marker(s) (in MC_{RF}).

1.1 Figure Legend

Figure S.1: Temporal alignment of data obtained from platform feedback and from motion capture system. In Panel A the blue lines represent the 3d coordinates of $\boldsymbol{p_c}$ (in S_{RF}). The vertical red lines represent the temporal window of selected data according to procedure descripts in paragraph S.1. The selected temporal aligned data are in black. In Panel B is showed the travelled distance (TD_{pc}^S) of $\boldsymbol{p_c}$ (in S_{RF}) used to identify the first peak of the sinewave signal (i_{start}^{Pc}). Panel C and D are in the same format of Panel A and B, but the data are related to a Marker (in MC_{RF}).
