# Supplementary figures and images for: Head-centric computing for vestibular stimulation under head-free conditions
Source: Front Bioeng Biotechnol. 2023 Dec 7;11:1296901. doi: 10.3389/fbioe.2023.1296901 (PMC10734306; doi:10.3389/fbioe.2023.1296901)

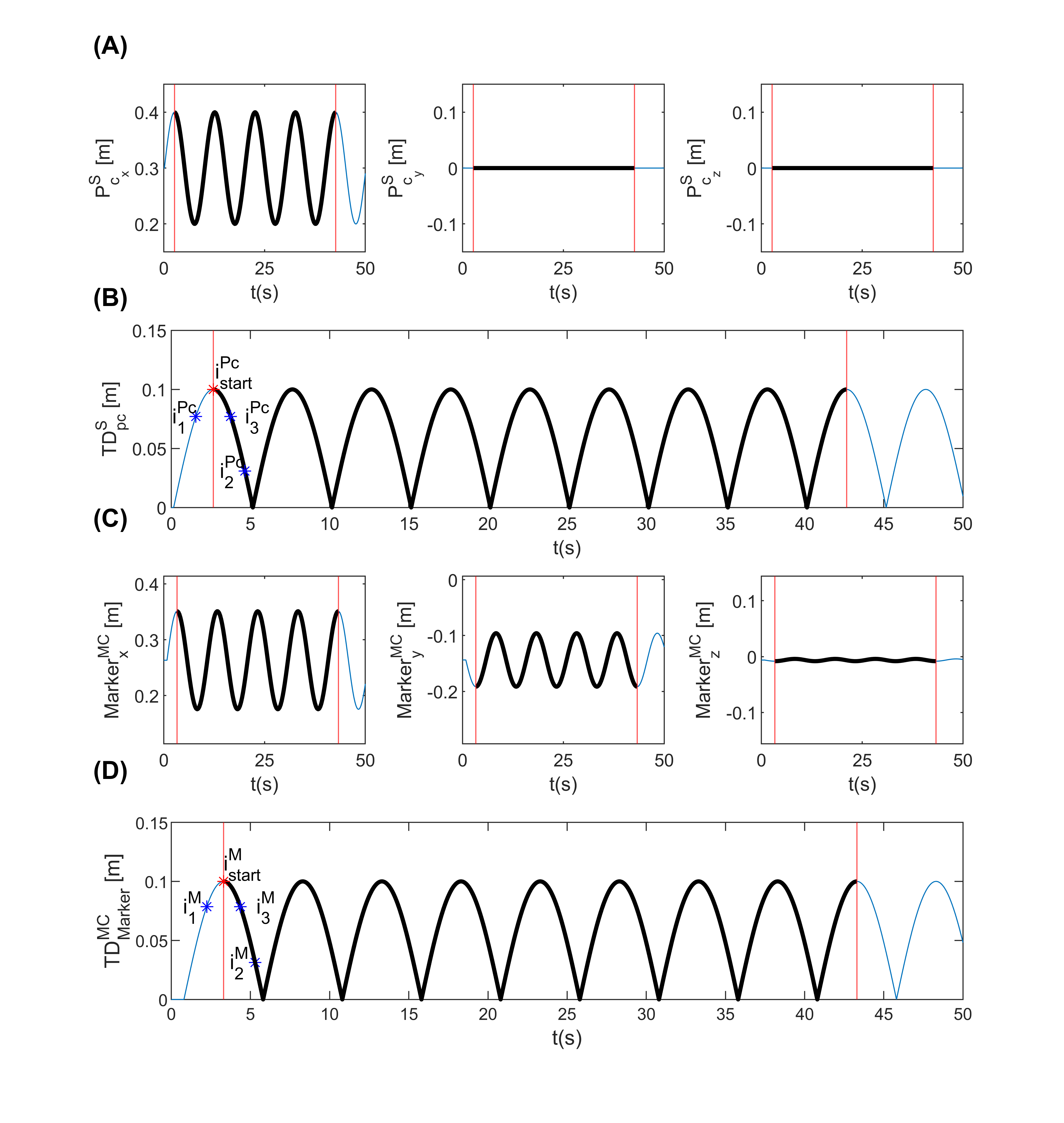

Supplement: Supplementary file 2 [file Image1.TIF]
